# T396I Mutation of Mouse *Sufu* Reduces the Stability and Activity of Gli3 Repressor

**DOI:** 10.1371/journal.pone.0119455

**Published:** 2015-03-11

**Authors:** Shigeru Makino, Olena Zhulyn, Rong Mo, Vijitha Puviindran, Xiaoyun Zhang, Takuya Murata, Ryutaro Fukumura, Yuichi Ishitsuka, Hayato Kotaki, Daisuke Matsumaru, Shunsuke Ishii, Chi-Chung Hui, Yoichi Gondo

**Affiliations:** 1 Mutagenesis and Genomics Team, RIKEN BioResource Center, Tsukuba, Ibaraki, Japan; 2 Department of Molecular Genetics, University of Toronto and Program in Developmental and Stem Cell Biology, The Hospital for Sick Children, Toronto, Ontario, Canada; 3 Department of Developmental Genetics, Institute of Advanced Medicine, Wakayama Medical University, Wakayama, Japan; 4 Laboratory of Molecular Genetics, RIKEN Tsukuba Institute, Tsukuba, Ibaraki, Japan; Schulze Center for Novel Therapeutics, Mayo Clinic, UNITED STATES

## Abstract

Hedgehog signaling is primarily transduced by two transcription factors: Gli2, which mainly acts as a full-length activator, and Gli3, which tends to be proteolytically processed from a full-length form (Gli3^FL^) to an N-terminal repressor (Gli3^REP^). Recent studies using a *Sufu* knockout mouse have indicated that Sufu is involved in regulating Gli2 and Gli3 activator and repressor activity at multiple steps of the signaling cascade; however, the mechanism of specific Gli2 and Gli3 regulation remains to be elucidated. In this study, we established an allelic series of ENU-induced mouse strains. Analysis of one of the missense alleles, *Sufu^T396I^*, showed that Thr^396 ^residue of Sufu played a key role in regulation of Gli3 activity. *Sufu^T396I/T396I ^* embryos exhibited severe polydactyly, which is indicative of compromised *Gli3* activity. Concomitantly, significant quantitative reductions of unprocessed Gli3 (Gli3^FL^) and processed Gli3 (Gli3^REP^) were observed *in vivo* as well as *in vitro*. Genetic experiments showed that patterning defects in the limb buds of *Sufu^T396I/T396I^* were rescued by a constitutive Gli3^REP ^allele (*Gli3^∆699^*), strongly suggesting that Sufu^T396I^ reduced the truncated Gli3 repressor. In contrast, Sufu^T396I^ qualitatively exhibited no mutational effects on Gli2 regulation. Taken together, the results of this study show that the Thr^396 ^residue of Sufu is specifically required for regulation of Gli3 but not Gli2. This implies a novel Sufu-mediated mechanism in which Gli2 activator and Gli3 repressor are differentially regulated.

## Introduction

Hedgehog (Hh) signaling is a key regulatory cascade that is involved in many developmental processes and diseases [1,2]. The Hh gradient is transduced through the activities of three Gli transcription factors: Gli1, Gli2, and Gli3, and directs pattern formation of tissues, including the embryonic neural tube and limb [3,4]. Genetic studies have shown that while *Gli1* is upregulated in a wide variety of tumors [5], *Gli1* KO mice show no morphological defects during development and are viable [6–8]. In contrast, mice lacking either *Gli2* (*Gli2* KO) or *Gli3* (*Gli3* ^*Xt*^) are embryonic lethal and exhibit distinct developmental defects; *Gli2* KO mice are compromised in ventral neural tube specification [7,9], and *Gli3* ^*Xt*^ mice develop exencephaly and polydactyly with a subtle patterning defect in intermediate region of the neural tube [6,10,11].

Both Gli2 and Gli3 have transcriptional activator and repressor domains in their C and N terminals, respectively [12,13]. The presence of Hh is considered to convert the latent full-length Gli2 and Gli3 (Gli^FL^) to a labile activator (Gli^ACT^) [14,15], which is subject to ubiquitin-mediated degradation [16,17]. In the absence of Hh signaling, Gli2 and Gli3 undergo sequential phosphorylation by multiple kinases and undergo limited proteolytic processing into a truncated N-terminal fragment, Gli^REP^ [14,18,19]. Although Gli2^REP^ only minimally contributes to Hh signal transduction [5], Gli3^REP^ is a potent negative regulator *in vivo*. The proteolytic processing of full-length Gli2 and Gli3 is mediated by Sufu [15,20,21]. In addition, Sufu antagonizes the complete degradation of Gli2^FL^ and Gli3^FL^ by antagonizing SPOP, which recruits ubiquitin ligase [16,17,20]. Notably, unlike Gli3^FL^, the processed Gli3^REP^ is not subject to further regulation by Sufu [15,20].

Previous studies of *Sufu* KO mice [22,23] showed that *Sufu* ^*−/−*^ embryos exhibited elevated Hh signaling and died at around E9 of gestation owing to severe patterning defects. Examination of neural tube patterning revealed that *Sufu* ^−/−^ embryos exhibited abnormal activation of Gli^ACT^ and a reduction in Gli^REP^ indicating that loss of *Sufu* affected both *Gli2* activator and *Gli3* repressor activity [24]. However, how this differential effect was achieved remained unclear.

To uncouple *Sufu-*mediated Gli2 and Gli3 regulation at the genetic and biochemical level, we generated an allelic series of *Sufu* point mutations using the RIKEN ENU-based gene-driven mutagenesis system [25]. These *Sufu* mutations ranged from null to hypomorphic alleles. Analysis of one such hypomorphic allele, *Sufu* ^*T396I*^, demonstrated a deficiency in Gli3 regulation, without qualitatively affecting the activities of Gli1 or Gli2. Further analysis of this missense mutation, presented in this report, revealed the novel function of the Thr^396^ residue of Sufu to be an essential role in Gli3 processing and stability. Analysis of the *Sufu* ^*T396I*^ mutant mouse line together with the allelic series of the *Sufu* gene should shed light on the elucidation of the molecular mechanism of *Gli* transcription factor regulation. All of the *Sufu* and *Smo* mutant lines established in this study are available from RIKEN BioResource Center.

## Results

### Identification of novel mutations in *Sufu*

**Fig 1:**
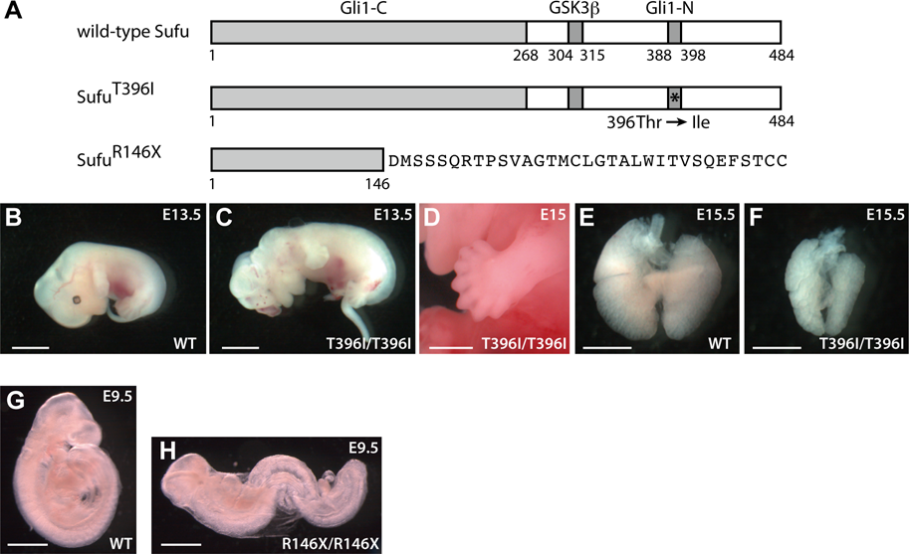
Identification of novel *Sufu* mutants. (**A**) Schematic diagrams of the mouse Sufu protein. The numbers refer to amino acid residues. Filled boxes indicate binding domains with GSK3β [21] and the C- and N-terminal regions of Gli1 [26]. An asterisk marks the position of a 1-base substitution that results in a change of Thr^396^ to Ile (T396I). *Sufu* ^*R146X*^ is a 1-base pair deletion in *Sufu*, leading to premature termination of the protein product after addition of an aberrant 33 amino acid stretch at the C terminal when translated. (**B, C**) Wild-type and *Sufu* ^*T396I/T396I*^ embryos at E13.5. Scale bar, 4 mm. (**D**) Limb phenotype of *Sufu* ^*T396I/T396I*^ at E15. Scale bar, 2 mm. (**E, F**) Lungs of wild-type and *Sufu* ^*T396I/T396I*^ at E15.5. Scale bars, 2 mm. (**G, H**) Wild-type and *Sufu* ^*R146X/R146X*^ embryos at E9.5. Scale bars, 1 mm. Homozygous embryos died at approximately E9.5 and exhibited an open brain and failure to undergo embryonic turning, characteristics identical to those reported in *Sufu* knockout embryos [22,23,51].

To identify functionally important residues in *Sufu*, we screened the ENU mutagenized mouse genome archive (details are described in the Materials and Methods in S1 File, and all identified mutations are listed in Table A in S1 File). Among the identified mutations, one point mutation, 1187C to T, leads to a substitution of threonine for isoleucine at Sufu residue 396 (T396I) (Fig. 1A). A previous study on cultured cells by Merchant et al. [26] indicated that Sufu residues 388–398 were highly conserved between invertebrates and vertebrates and played an essential role in mediating the physical interaction of Sufu with the N-terminal region of Gli1. On the basis of these findings, we anticipated that the T396I mutation would compromise Sufu–Gli1 interaction. However, analysis of *Sufu* ^*T396I/T396I*^ embryos revealed that the mutants developed phenotypes similar to those of mice lacking not *Gli1* but *Gli3* (*Gli3* ^*Xt/Xt*^) [6,10]. The *Sufu* ^*T396I/T396I*^ mice died at E14–18 with peripheral edema, hemorrhage, and severe morphological defects including exencephaly and polydactyly (Fig. 1B–D). Craniofacial and limb defects in *Sufu* ^*T396I/T396I*^ were indicative of compromised *Gli3* function [5]. In addition, *Sufu* ^*T396I/T396I*^ developed abnormal lungs that were small and thickened but had the correct number of lobes (Fig. 1E, F), one of the characteristics similar to that observed in *Gli3* ^*Xt/Xt*^ [27]. These phenotypes were strikingly different from the early lethality and elevated Hh signaling previously reported for *Sufu* KO [22,23] and recapitulated in our analysis of *Sufu* ^*R146X/R146X*^ null mutants established in the present study (Fig. 1A, G, H and S1 Fig.). These findings suggested that the Thr^396^ residue of Sufu is critical for the regulation of Gli3 repressor; we tested this hypothesis with further *in vitro* and *in vivo* studies as described below.

### *Sufu* ^*T396I/T396I*^ showed drastic reduction of Gli3^FL^ and Gli3^REP^ levels

**Fig 2:**
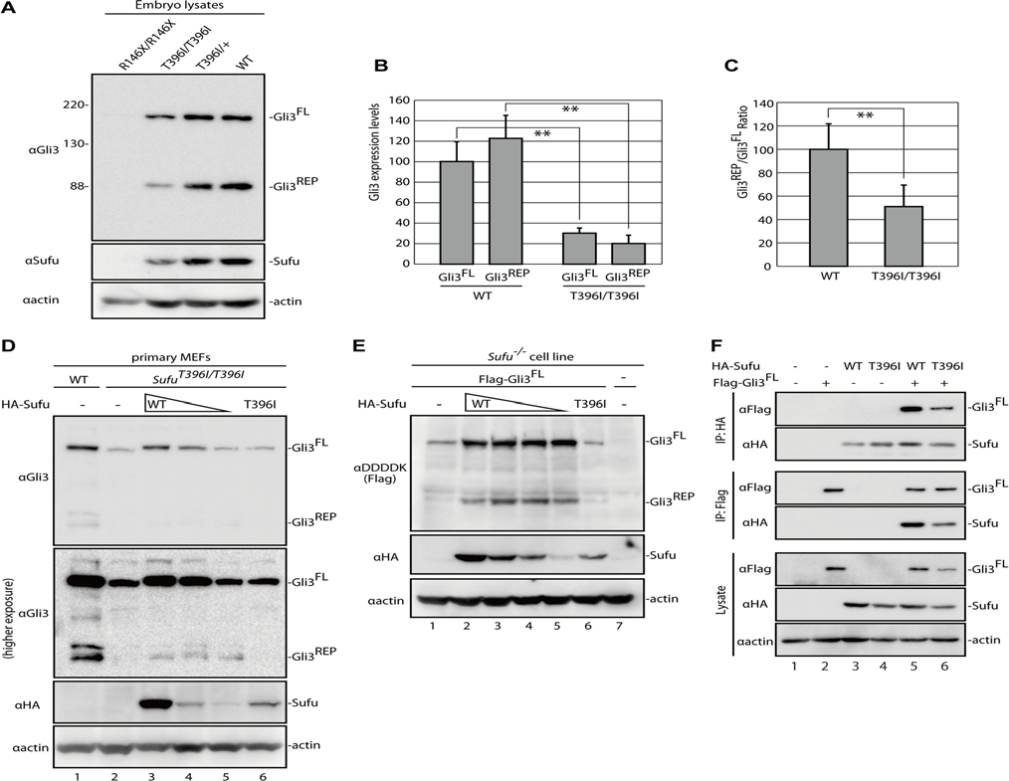
Sufu^T396I^ does not stabilize Gli3^FL^ protein and reduces the processing of Gli3^FL^. (**A**) Western blotting of lysates prepared from *Sufu* ^*R146X/R146X*^ at E9.5 and *Sufu* ^*T396I/T396I*^, *Sufu* ^*T396I/+*^, and wild-type embryos at E10.5 with anti-Gli3, anti-Sufu, and anti-actin antibodies. Each image presented in the Fig. is a representative of independent triplicated experiments. The full gel images are shown in S6A Fig. (**B, C**) Relative expression of Gli3^FL^ (B) and Gli3^REP^ (B and C). Western blotting was performed two times using lysates prepared from five wild-type and five *Sufu* ^*T396I/T396I*^ embryos at E10.5 (S2A Fig.). Expression levels were quantified from the band intensity shown in S2A Fig. as relative values of the Gli3^FL^/actin and Gli3^REP^/actin expression ratios (B) and the direct ratio of Gli3^REP^/Gli3^FL^ (C). (**) p < 0.01, two-tailed Student’s t-test. Error bars indicate the standard deviations. (**D**) Western blotting of cell lysates from wild-type and *Sufu* ^*T396I/T396I*^ MEFs with indicated antibodies. The *Sufu* ^*T396I/T396I*^ MEFs were electroporated with 10.0, 1.0, or 0.1 μg of the HA–Sufu construct (lane 3, 4, and 5, respectively), or 10 μg of the HA–Sufu^T396I^ construct (lane 6). The mobilities on SDS-PAGE of the wild-type Sufu (lane 3–5) and Sufu^T396I^ (lane 6) are identical. The complete gel images are shown in S6B Fig. This image is representative of two independent experiments. **(E)** Western blotting of cell lysates from *Sufu* ^*−/−*^ cells with indicated antibodies. The *Sufu* ^*−/−*^ cells were electroporated with a mixture of the Flag–Gli3 construct (6 μg) and the HA–Sufu construct (4.00, 1.33, 0.44, or 0.15 μg) for the wild-type Sufu cotransfection (lane 2 to 5, respectively), or a mixture of the Flag–Gli3 construct (6 μg) and the HA–Sufu^T396I^ construct (4 μg) for mutant Sufu cotransfection (lane 6). The complete gel images are shown in S6C Fig. This image is representative of two independent experiments. **(F)** Western blotting of immunoprecipitates or lysates from 293T cells transfected with expression constructs as indicated at the top. The complete gel images are shown in S6D Fig. This image is representative of two independent experiments.

To determine how Sufu^T396I^ regulates Gli3 activity, we evaluated Gli3 expression in *Sufu* ^*T396I/T396I*^ embryos. Western blot analysis showed significant reduction of both Gli3^FL^ and Gli3^REP^ protein in *Sufu* ^*T396I/T396I*^ embryos at E10.5 (Fig. 2A–C and S2A Fig.). The expression level of *Gli3* mRNA, assessed by RT-PCR, was unchanged in these mutants (S2B Fig.); this suggests that Thr^396^ is specifically required to stabilize Gli3^FL^. We also observed a decrease in Gli3^REP^. Because it is a product of Gli3^FL^ processing, it is natural to observe a reduction in Gli3^REP^ when Gli3^FL^ levels are low. However, the ratio of Gli3^REP^ to Gli3^FL^ was approximately 50% lower in *Sufu* ^*T396I/T396I*^ than in wild-type embryos (Fig. 2C). This finding suggested that Thr^396^ is required not only to stabilize Gli3^FL^ but also for its proteolytic processing to Gli3^REP^.

To identify the specific effect of the T396I mutation on the regulation of Gli3, we investigated how *Sufu* ^*T396I*^ affects the level of endogenous Gli3 in primary mouse embryonic fibroblasts (MEFs). We showed that overexpression of wild-type *Sufu*, but not *Sufu* ^*T396I*^, can stabilize endogenous Gli3^FL^ in *Sufu* ^*T396I/T396I*^ primary MEFs (Fig. 2D and Table B in S1 File). Similarly, only wild-type Sufu, but not *Sufu* ^*T396I*^, can stabilize the level of Flag–Gli3 overexpressed in immortalized *Sufu* ^*−/−*^ cells [16] (Fig. 2E and Table B in S1 File). Notably, even high levels of Sufu^T396I^ could not stabilize Gli3^FL^ (Fig. 2D, compare lane 6 with lane 4; Fig. 2E, compare lane 6 with lane 5). In addition to compromised Gli3^FL^, Gli3^REP^ was barely detectable in *Sufu* ^*T396I/T396I*^ MEFs. Although overexpression of wild-type Sufu promoted the production of a small amount of Gli3^REP^, overexpression of Sufu^T396I^ had no effect, despite equal amounts of Gli3^FL^ (Fig. 2D, higher exposure, compare lane 6 with lane 5), indicating that Thr^396^ is required for both Gli3^FL^ stabilization and processing.

We found that expression of the Sufu protein was also reduced in *Sufu* ^*T396I/T396I*^ (Fig. 2A and 5A). It must be noted that the expression of *Sufu* mRNA in *Sufu* ^*T396I/T396I*^ embryos was comparable to that in wild-type embryos (S3A Fig.). The instability of Sufu^T396I^ was also shown *in vitro* (S3B Fig.). When the level of Sufu protein was examined after cycloheximide treatment, the Sufu^T396I^ protein disappeared more rapidly than wild-type Sufu.

Although Sufu residues 388–398 are required for Sufu-Gli1 interaction, substitution of Thr^396^ to alanine or aspartate did not affect the physical binding of Sufu to Gli1 [26]. To determine whether the Thr^396^ residue is important for regulating the physical interaction of Sufu^T396I^ with the other Gli transcription factors, we performed immunoprecipitation analysis in cultured cells. We found that Sufu^T396I^ coimmunoprecipitated with Gli3^FL^, as shown in Fig. 2F, lane 6. This result suggests that Sufu^T396I^ still retains the ability for the physical interaction with Gli3^FL^, whereas the Thr^396^ residue is evidently required for stabilization and processing of Gli3.

### Gli3^REP^ activity was reduced in the *Sufu* ^*T396I/T386I*^ limb development

To evaluate the effect of *Sufu* ^*T396I*^ *in vivo*, we turned to the embryonic limb, given that Gli3^REP^ plays a critical role in the anteroposterior (AP) pattern of the digits [4], and *Sufu* ^*T396I/T396I*^ mutants develop severe polydactyly with unpatterned digits, associated with compromised Gli3 function (Fig. 1C, D).

**Fig 3:**
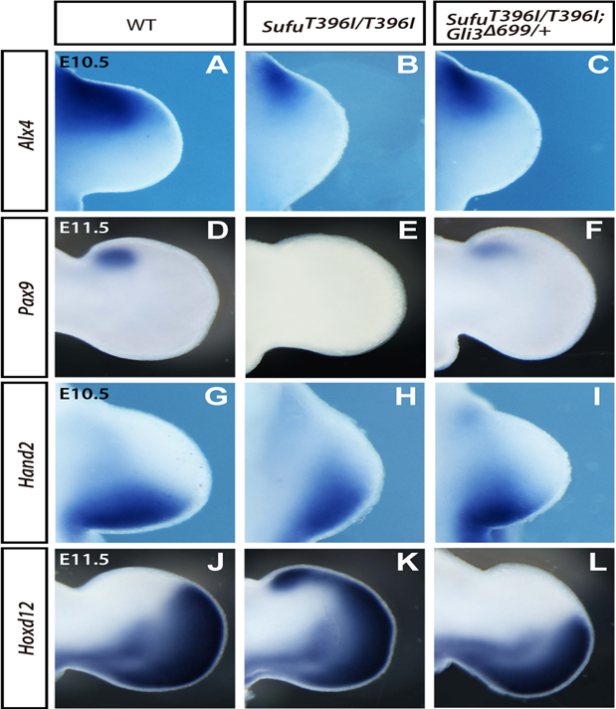
*Gli3* ^*∆699*^ suppresses the A-P polarity defects in the *Sufu* ^*T396I/T396I*^ limb buds. (**A–L**) Expression of *Alx4* (A–C), *Pax9* (D–F), *Hand2* (G–I), and *Hoxd12* (J–L) of wild-type (A, D, G and J), *Sufu* ^*T396I/T396I*^ (B, E, H, and K), and *Sufu* ^*T396I/T396I*^; *Gli3* ^*∆699/+*^ (C, F, I, and L) by RNA *in situ* hybridization at indicated stages. Genotypes are indicated at the top. Limb buds are oriented with the anterior to the top.

It is known that mutual antagonistic interactions between *Gli3* and *Hand2* are required to establish early AP patterning in the limb and correct expression of downstream genes including *Alx4* and *Pax9* in the anterior mesenchyme [28,29] and *Hand2* and *Hoxd12* in the posterior mesenchyme [30]. Analysis of *Sufu* ^*T396I/T396I*^ limbs revealed decreased expression of Gli3 target genes *Alx4* and *Pax9* in the anterior of the limb (Fig. 3A, B, D, E) and ectopic expression of posterior genes *Hoxd12* and *Hand2* throughout the limb bud mesenchyme (Fig. 3G, H, J, K). These phenotypes were consistent with compromised Gli3^REP^ function [31–33]. To determine whether digit defects in *Sufu* ^*T396I/T396I*^ are due to impaired Gli3^REP^ activity, we forced expression of a constitutive Gli3^REP^ allele (*Gli3* ^*∆699*^), which produces a truncated protein due to a premature termination codon [34], in the *Sufu* ^*T396I/T396I*^ background. We showed that expression of *Gli3* ^*∆699*^ was sufficient to rescue the expression of *Alx4* and *Pax9* (Fig. 3C, F) and restore polarized expression of *Hand2* and *Hoxd12* (Fig. 3I, L). These findings indicate that the patterning defects in the *Sufu* ^*T396I/T396I*^ limb may be attributed to compromised Gli3^REP^ activity.

### *Sufu* ^*T396I/T396I*^ embryos lost Gli3^REP^ activity but retained Gli2 activity

**Fig 4:**
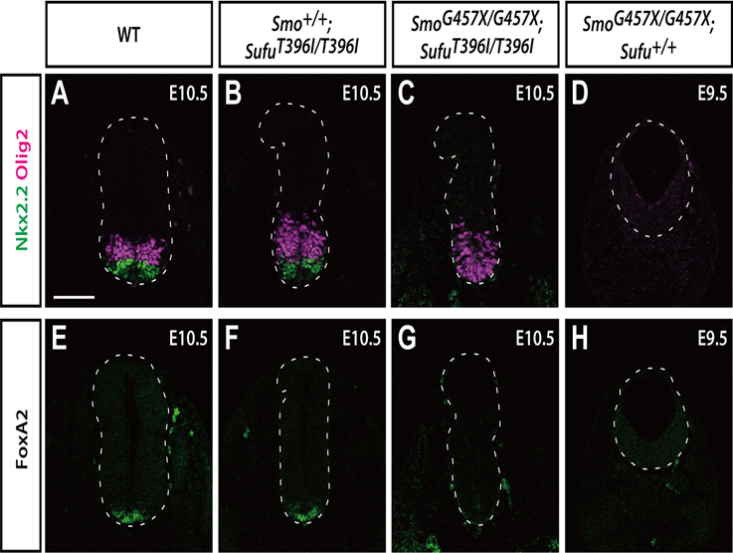
*Sufu* ^*T396I/T396I*^ shows reduction of Gli3 activity but not Gli2 activity. (**A–H**) Immunofluorescence images of transverse sections at thoracic level. Sections from wild-type (A and E), *Sufu* ^*T396I/T396I*^ (B and F), and *Smo* ^*G457X/G457X*^; *Sufu* ^*T396I/T396I*^ (C and G) embryos at E10.5 and *Smo* ^*G457X/G457X*^ embryos (D and H) at E9.5 were immunostained with anti-Olig2 (A–D, magenta), anti-Nkx2.2 (A–D, green), and anti-FoxA2 (E–H, green) antibodies. Dashed lines outline the neural tubes. Scale bar, 100 μm. Images with nuclear staining are shown in S7 Fig.

To investigate whether the *Sufu* ^*T396I*^ mutation also affects Gli^ACT^ as well as Gli^REP^, we analyzed development of the ventral neural tube, given that the generation of neural progenitor cells along its D-V axis is dependent on the Shh signaling gradient and Gli^ACT^ activity [3]. In particular, activation of Gli2 is essential to generate floor plate (FoxA2 positive cells) and p3 progenitors (Nkx2.2 positive cells) in response to high Shh signaling (Fig. 4A, green and 4E) [35]. Motor neuron progenitor cells (Olig2 positive cells) are generated dorsal to p3 progenitors in response to intermediate Shh signaling and a balance between Gli2^ACT^ and Gli3^REP^ (Fig. 4A, magenta) as has been reported before [11,36]. Previous studies [37–39] showed that loss of *Smo*, a core pathway regulator upstream of Sufu, leads to a decreased level of Gli^ACT^ and excess Gli3^REP^, resulting in a failure of ventral specification. A double homozygous mutation of *Smo* and *Gli3* restores expression of Olig2 in the ventral neural tube [36,39], indicating that Gli3^REP^ represses Olig2 in the *Smo* ^*−/−*^ genetic background.

Based on these previous findings, we evaluated the marker gene expression of Olig2, Nkx2.2, and FoxA2 in a double mutation of *Sufu* ^*T396I/T396I*^ and *Smo* ^*G457X/G457X*^. The *Smo* ^*G457X*^ mutation is a null allele, as also established in the present study (Table A in S1 File and S4 Fig.). As in *Smo* ^*−/−*^ embryos [39–41], the expression of FoxA2, Nkx2.2, and Olig2 was completely repressed in the ventral neural tube of *Smo* ^*G457X/G457X*^ (Fig. 4D, H). In contrast, *Sufu* ^*T396I/T396I*^; *Smo* ^*G457X/G457X*^ double mutants restored expression of Olig2 at the ventral midline (Fig. 4C, magenta), although expression of Nkx2.2 and FoxA2 was still inhibited (Fig. 4C, green and 4G). This expression pattern is identical to one previously reported in *Smo* ^*−/−*^; *Gli3* ^*Xt/Xt*^ double mutants [36,39]. This finding indicated that Thr^396^ is critical for promoting the activity of Gli3^REP^.

Notably, the expression of Nkx2.2 and FoxA2, although still absent in *Sufu* ^*T396I/T396I*^; *Smo* ^*G457X/G457X*^, appears normal in *Sufu* ^*T396I/T396I*^ (Fig. 4B, green and 4F). It is well established that expression of Nkx2.2 and FoxA2 depends on Gli2^ACT^. This dependence indicates that *Sufu* ^*T396I/T396I*^ does not activate *Gli2* independent of *Smo* and suggests that Thr^396^ is not required for Gli2 regulation.

### Thr^396^ is not required for regulation of Gli activators

**Fig 5:**
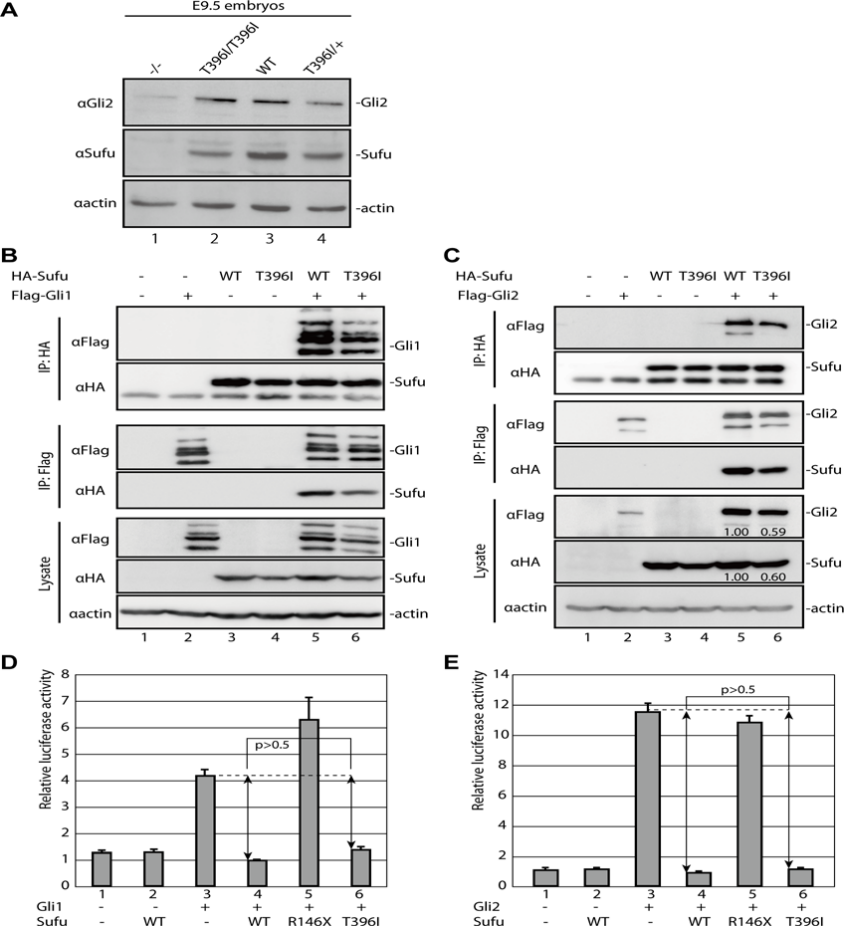
Sufu^T396I^ can regulate Gli activator function. (**A**) Western blotting of Gli2 from embryo lysates at E9.5. Genotypes and antibodies are indicated at the top and left, respectively. The image presented in the Fig. is representative of independent triplicated experiments. The broad gel images are shown in S5A Fig. and relative expression of Gli2 is shown in S5B Fig. (**B, C**) Western blotting of immunoprecipitates or lysates from 293T cells transfected with expression constructs as indicated at the top. Antibodies used for immunoprecipitation and western blotting are indicated to the left. The complete gel images are shown in S5C and D Fig. Gli1 protein appears to show multiple bands; the reason for this is unknown [42] [46]. Relative band intensities of lane 5 and 6 (C) are shown. It is note worthy that the expression levels of Gli2 were proportional to the amount of Sufu irrespective of either wild-type or T396I substitutions. (**D, E**) Luciferase reporter assay in 3T3 transfected with expression constructs as indicated in figures. Error bars indicate standard deviations. In our experimental settings, we observed approximately only 4-fold induction of luciferase reporter activity by Gli1 expression, as had been observed previously [46].

To assess the effect of T396I on Gli2 stability and regulation, we compared the levels of full-length Gli2 protein in wild-type and *Sufu* ^*T396I/T396I*^ embryos. We showed that expression of Gli2 was comparable in the point mutant and the wild-type embryos (Fig. 5A, lane 2 and S5A, B Fig.). In contrast, *Sufu* ^*−/−*^ embryos were severely depleted in full-length Gli2 protein (Fig. 5A, lane 1). This is consistent with previous studies [16,20], in which Sufu is shown to be required to stabilize Gli2. Thus, although Thr^396^ is critical for stabilization of full-length Gli3, it is dispensable for stabilization of Gli2.

To further investigate how Thr^396^ affects Gli1 and Gli2 function, we overexpressed Gli2 together with wild-type Sufu or Sufu^T396I^ in cultured cells (Fig. 5C, lysate). We detected no differences between wild-type Sufu and Sufu^T396I^ with respect to stabilization of Gli2 protein, indicating that the activities of wild-type Sufu and Sufu^T396I^ were qualitatively equivalent on Gli2 stabilization both *in vitro* and *in vivo*. In addition, consistent with a previous study [26], we showed that Sufu^T396I^ retained the ability to interact with Gli1 and Gli2 in immunoprecipitation experiments to similar or somewhat reduced extent as wild-type Sufu (Fig. 5B, C and Table B in S1 File).

Finally, it is known that Sufu normally sequesters Gli1 and Gli2 to the cytoplasm [42–46]. Consequently, Sufu overexpression inhibits Gli1 and Gli2 trafficking into the nucleus and transcriptional activity. This activity can be detected by assessing the transcriptional activation of a reporter gene, 8xGliBS, controlled by Gli1 and Gli2 [13,42]. To determine whether Sufu^T396I^ had a similar repressive activity, we performed a reporter assay in 3T3 cells, a Hh-responsive cell line. We found that Sufu^T396I^ was able to repress the transcriptional activation of the 8xGliBS reporter by both Gli1 (Fig. 5D) and Gli2 (Fig. 5E), similar to wild-type Sufu. In addition, a titration assay showed the Gli1 and Gli2 activities were proportional to the amount of Sufu irrespective of either wild-type or the T396I substitution (S8 Fig.). Thus, the repressor activities of wild-type Sufu and Sufu^T396I^ were qualitatively equivalent on Gli1 and Gli2 regulations. This finding strongly suggested that the Thr^396^ residue of Sufu is not required for the stabilization or transcriptional activity of Gli1 and Gli2.

## Discussion

In this work, we showed that *Sufu* ^*T396I*^ missense mutant mice uncouple molecular regulation of Gli3 from Gli1/2. This mutant was generated as part of an allelic series using the RIKEN ENU gene-driven mutagenesis system. The results presented here show that generation of point mutations in critical regulatory residues make it feasible to connect biochemical interactions between key molecules in the Hh signaling pathway with the genetics of the system.

### Phenotype of *Sufu* ^*T396I/T396I*^ is attributed to a loss of Gli3 regulation

We conclude that the alteration of Sufu function due to T396I substitution rather than the reduction of the Sufu amount per se, is a major primary cause of morphological defects in *Sufu* ^*T396I/T396I*^. This is because Sufu^T396I^ is not able to stabilize Gli3^FL^ in primary MEFs of *Sufu* ^*T396I/T396I*^ embryos as well as in an immortalized *Sufu* ^*−/−*^ cell line, in which the expression level of Sufu^T396I^ is higher than that of wild-type Sufu. In addition, overexpression of Sufu^T396I^ did not increase Gli3^REP^ in the *Sufu* ^*T396I/T396I*^ MEFs. Thus, Sufu^T396I^ was qualitatively less efficient both for stabilizing Gli3^FL^ and for mediating proteolytic processing of Gli3^FL^ to Gli3^REP^ than wild-type Sufu.

### Distinct regulation of Gli activator and Gli3 repressor by Sufu

Recent reports [15,16,20] have shown that Sufu and SPOP, a substrate-binding adaptor for Cul3-based E3 ubiquitin ligase, competitively bind Gli2^FL^ and Gli3^FL^ and oppose their activity on each other. In this context, Sufu protects Gli2^FL^ and Gli3^FL^ from proteasome degradation by ubiquitin ligase and regulates Gli protein levels. In addition, Sufu mediates proteolytic processing of Gli2 and Gli3 by β-TrCP/SCF-type E3 ubiquitin ligase to generate the N-terminal fragment, Gli^REP^ [19,47]. Thus, Sufu independently regulates two proteasome-related processes that control both the amounts and activities of Gli2 and Gli3. However, whether Sufu regulates Gli2 and Gli3 through a common action or different actions remains unknown. Our observations that Sufu^T396I^ was able to control Gli2 but not Gli3 are strong evidence of distinct regulatory mechanisms of Gli activator and repressor. Namely, one is the Thr^396^-independent regulation of Gli2 and the other is the Thr^396^-dependent regulation of stability and proteolytic processing of Gli3.

### Three-dimensional structure of Sufu and its interaction with Gli

Recent studies have solved the 3D structures of full-length human Sufu alone and in a complex with a Gli-derived peptide containing the N-terminal SYGHL Gli motif [48,49]. They showed that Sufu consists of N- and C-terminal globular domains with a short linker and displays “open” and “closed” conformations. Activation of Hh signaling is associated with promotion of the open form of Sufu and dissociation of Gli, whereas inhibition of the signaling is associated with promotion of the closed form of Sufu and Gli binding. The Thr^396^ residue of Sufu is located on strand β-13 that juxtaposes strand β-9, which mediates Gli-binding [48]. Thus, substitution of Thr^396^ would not directly affect interaction between strand β-9 of Sufu and the SYGHL motif of Gli. In addition, a previous study in cultured cells indicated that substitution of Thr^396^ to alanine (T396A) or aspartic acid (T396D) did not affect the physical interaction of Sufu with Gli1 [26]. In the present study, we have also found that Sufu^T396I^ still retains the ability to physically interact with Gli1, Gli2, and Gli3 with similar or somewhat reduced extent to wild-type Sufu. Thus, structural insight and molecular biology support our hypothesis that substitution of Thr^396^ does not affect the gross structure that constitutes the Gli binding site and mediates direct physical interaction with Gli.

In contrast to these structural studies, previous deletion mapping approaches indicated that the N- and C-terminal fragments of Sufu were able to separately bind Gli1 and Gli2 (Fig. 1A) [26,46]. In particular, substitution of residues 388–398 of Sufu with an alanine homopolymer disrupted its physical interaction with an N-terminal fragment of Gli1. This observation implies an interaction(s) between Gli and the C-terminal region of Sufu, which includes Thr^396^, in addition to the above mentioned physical interaction between the SYGHL motif of Gli and strand β9 of Sufu. Given our findings, this possible interaction is likely to be associated with precise regulation specific to Gli3 but not Gli2.

### Thr^396^ is essential for regulation of Gli3 activity

A recent study has shown that Sufu recruits GSK3β and forms the trimolecular complex Gli3/Sufu/GSK3β [21]. In the absence of Hh, GSK3β in the trimolecular complex efficiently phosphorylates Gli3^FL^, leading to Gli3 ubiquitination and then proteolytic processing to generate Gli3^REP^ [18]. The GSK3β-binding region of Sufu is defined as the medial region between residues 304 and 315 (Fig. 1A) [21], which is part of the “intrinsically disordered region” (IDR) indicated by the 3D structure of full-length human Sufu [48,49]. IDR is a flexible loop with no fixed structure and appears to shield the Gli-binding surface of Sufu in response to upstream signaling. Thus, substitution of Thr^396^ may indirectly affect the flexibility of IDR, leading to failure of the trimolecular complex formation with GSK3β or regulation of the GSK3β activity.

Embryonic lethal mutations in *Drosophila* enabled the elucidation of molecular pathways and key elements in early embryogenesis [50]. The allelic series of target genes in a specific gene network, such as Hh signaling as described in this study, should prime analogous molecular approaches for the elucidation of molecular mechanisms in mammalian development.

## Materials and Methods

### Mutation screening

ENU mouse mutagenesis and gene-driven screening were described in Materials and Methods in S1 File in details. The primers used for the mutation screening are listed in Table C in S1 File. All the identified ENU-induced mutations in the *Sufu* and *Smo* genes are summarized in Table D in S1 File.

### Mice

All animal work was conducted according to the protocols and guidelines approved by the ethics committee of RIKEN Tsukuba Institute (Permit number: 14–012). The animals were sacrificed by cervical dislocation. *Sufu* ^*T396I*^, *Sufu* ^*R146X*^, and *Smo* ^*G457X*^ lines were revived from ENU Mutant Mouse Library (reviewed by Gondo) [25] by a conventional IVF technique after the mutation screening. Congenic lines of these three mutations were established by breeding more than 10 generations of backcrosses to C57BL/6J strain. Details of genotyping are described in Materials and Methods in S1 File with all the used primer information in Table C, E, and F in S1 File. The *Gli3* ^*∆699*^ mouse line [34] and the *Sufu* ^*−/−*^ mouse line [51] were maintained on CD1 background. ENU mouse mutant lines are available from RIKEN BioResource Center.

### Molecular biology and constructs

*Sufu* cDNA [42] and mouse *Gli3* cDNA were N-terminally tagged with HA and 3xFlag, respectively. Point mutations of *Sufu* ^*R146X*^, *Sufu* ^*T396I*^, and *Smo* ^*G457X*^ were introduced with the QuickChangeII Site-Directed Mutagenesis Kit (Agilent Technologies). All constructs were verified by direct sequencing. Plasmids expressing Flag–Gli1and Flag–Gli2, were previously described [42].

### Cell culture and transfection

Cells were grown in DMEM supplemented with 10% fetal bovine serum, penicillin, and streptomycin. NIH3T3 and 293T cells were transfected with HilyMax transfection reagent (Dojindo Laboratories). MEFs were prepared from WT and *Sufu* ^*T396I/T396I*^ embryos at E13.5 according to standard techniques [23] with the above medium. Because the *Sufu* ^*−/−*^ cells and MEFs had a low transfection efficiency by lipofection, the cells were electroporated with a MEF2 Nucleofector kit (Lonza) using an A-23 program according to the manufacturer’s instructions. The total amount of DNA for electroporation was adjusted to 10 μg by addition of the pUC vector as a control plasmid.

### Luciferase reporter assay

Reporter assay was performed by transfecting 250 ng of reporter vector with firefly luciferase under the control of eight Gli binding sites [13], 50 ng of pRLTK (Promega) as a Renilla control, and 100 ng of Gli1 or Gli2 and 100 ng of Sufu expression constructs, into NIH3T3 in a 24–well plate. After confluence was reached, the culture medium was replaced with a low-serum (0.5%) medium and cultured for an additional 24 h. Cells were harvested and luciferase activity was measured with a Dual Luciferase Reporter Assay System (Promega) on an ARVO MX 1420 Multilabel Counter System (Perkin Elmer). Each assay was calculated from triplicate wells, and at least three independent assays were performed. Data from a representative experiment are shown in the figures. The statistical analysis in Fig. 5 was conducted as follows. Firstly, the effects of the Sufu repressor activity on Gli1 and Gli2 were calculated by:

The repressor activity of the wild−type Sufu=lane4–lane3=Rep(wild) (1)

The repressor activity of the T396I Sufu=lane6–lane3=Rep(T396I) (2)

Then, the difference of the repressor activities between wild-type and T396I Sufu, namely Rep(wild)—Rep(T396I), was subjected to two-tailed Student’ t-test.

### Coimmunoprecipitation

Twenty-four hours after transfection, 293T cells were lysed in a lysis buffer (0.1 mM Tris-HCl (pH 7.5), 0.3 M NaCl, 2% Nonidet P-40, 2 mM EDTA, and Complete Mini protease inhibitor cocktail (Roche) for 10 min on ice. Following centrifugation, cleared lysates were incubated with anti-HA magnetic beads (Medical & Biological Laboratories) or anti-FLAG M2 magnetic beads (Sigma) for 2 h at 4°C with constant nutation. Beads were washed three times with lysis buffer and mixed with SDS loading buffer. Supernatants were analyzed by western blotting.

### Western blotting

Dissected embryos were lysed in lysis buffer for 10 min on ice. Following centrifugation, the protein concentration of cleared lysates was determined by Quick Start Bradford protein assay (BioRad). Equal amount of protein was resolved on 6% SDS-PAGE gels. The transferred membrane was immunoblotted with the SNAP i.d. system according to the manufacturer’s instructions (Merck Ltd.). Chemiluminescence images were captured with a LAS 3000 imaging system (GE Healthcare) except for Fig. 5A and S5A Fig. Western blotting with Gli2 antibody was performed by standard procedures, and the membrane was exposed to an X-ray film for Fig. 5A and S5A Fig. Band intensity was measured with Image Quant TL software (GE Healthcare). The primary and secondary antibodies used were rabbit anti-Gli2 [16], goat anti-Gli3 (AF3690, R&D Systems), rabbit anti-Sufu (Fig. 5A and S5A Fig.) [52], rabbit anti-Sufu (except Fig. 5A and S5A Fig.) (ab28083, Abcam), rabbit anti-HA (ab9110, Abcam), mouse anti-FLAG M2 (Sigma), mouse anti-DDDDK-tag (MBL), mouse IgM anti-actin (Ab-1, Calbiochem), donkey anti-goat HRP (Molecular Probes), goat anti-rabbit HRP (Molecular Probes), goat anti-mouse HRP (Molecular Probes), and goat anti-mouse IgM HRP (Southern Biotech) antibodies.

### Immunohistochemistry and *in situ* hybridization

Immunohistochemistry was performed with 7 μm paraffin embedded sections as previously described [53]. The antibodies used were Pax7 (bio reactor, 1/500), Nkx6.1 (concentrate, 1/6000), Nkx2.2 (concentrate, 1/600), and FoxA2 (concentrate, 1/100) from Developmental Studies Hybridoma Bank, and Olig2 (AB9610, 1/6000) from Millipore. Images were acquired with an LSM510 Laser Scanning Microscope (Zeiss). Whole mount *in situ* hybridization was performed with digoxigenin-labeled riboprobes against *Hand2*, *Alx4*, *Hoxd12*, and *Pax9* as described [7].

## Supporting Information

S1 Fig *Sufu* ^*R146X*^ is a null allele of *Sufu*. (TIF)

S2 Fig Expression levels of both Gli3^FL^ and Gli3^REP^ are reduced in *Sufu* ^*T396I/T396I*^ embryos. (TIF)

S3 Fig Stability of the Sufu^T396I^ protein is reduced. (TIF)

S4 Fig *Smo* ^*G457X*^ is a null allele of *Smo*. (TIF)

S5 Fig Sufu^T396I^ is able to stabilize Gli2 and interact with Gli1 and Gli2. (TIF)

S6 Fig Full gel images of western blotting shown in Fig. 2. (TIF)

S7 Fig Nuclear staining of the neural tubes shown in Fig. 4. (TIF)

S8 Fig Qualitatively equivalent activities of wild-type Sufu and Sufu^T396I^ on Gli1 and Gli2 regulations. (TIF)

S1 File Materials and Methods, Table A-F, References, S1–S8 Fig. Legends. Table A: Summary of identified mutations for the *Sufu* and *Smo* genes. Table B: Quantification of band intensity from Western blotting. Table C: Primer sequences for mutation screening. Table D: Summary of TGCE screening in the *Sufu* and *Smo* genes. Table E: Primers used for genotyping by pyrosequencing. Table F: Taqman probes and primer sequences for genotyping.
(DOCX)
