# Supplementary figures and images for: T396I Mutation of Mouse Sufu Reduces the Stability and Activity of Gli3 Repressor
Source: PLoS One. 2015 Mar 11;10(3):e0119455. doi: 10.1371/journal.pone.0119455 (PMC4356511; doi:10.1371/journal.pone.0119455)

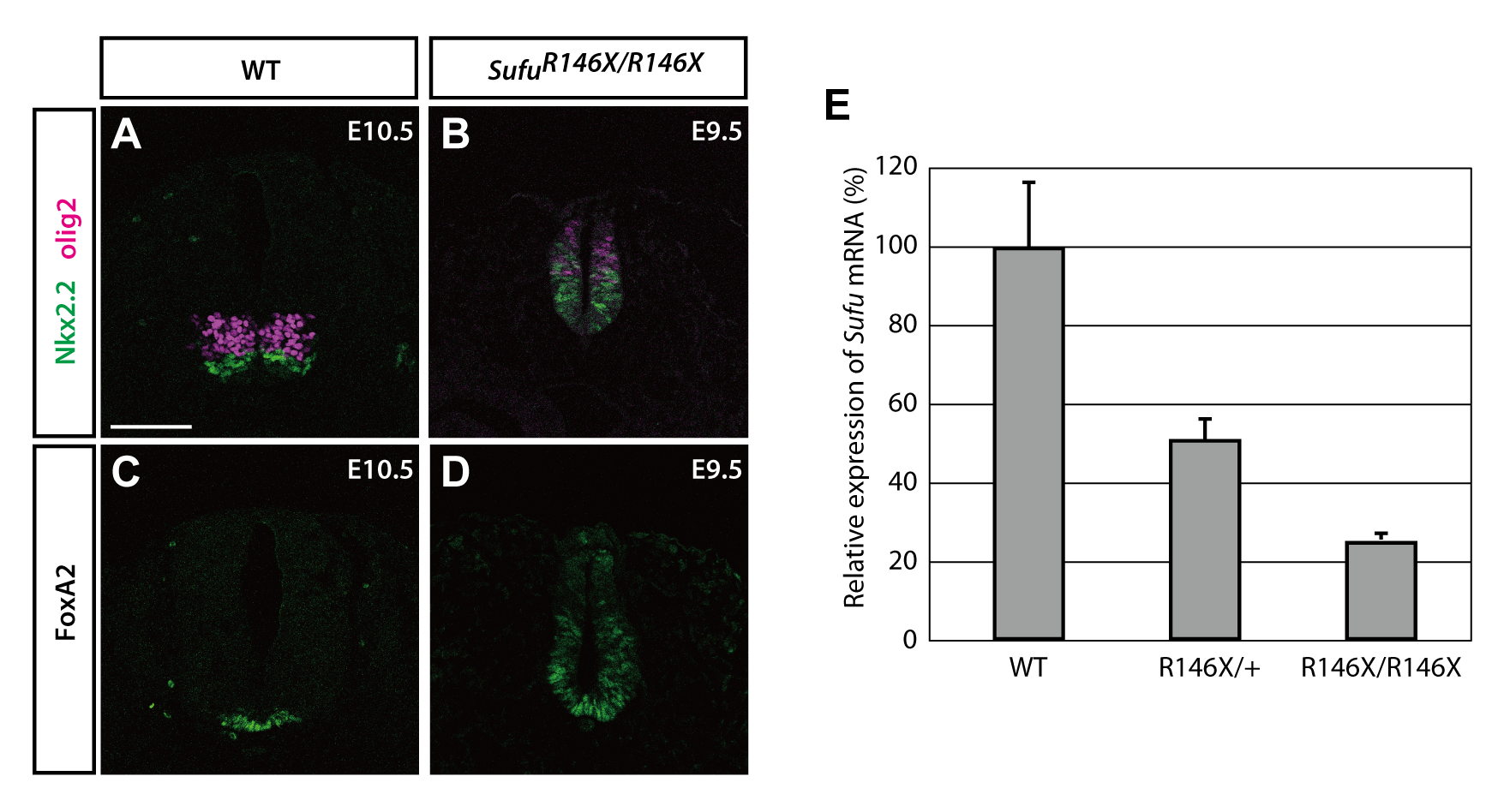

Supplement: S1 Fig — (TIF) [file pone.0119455.s001.tif]

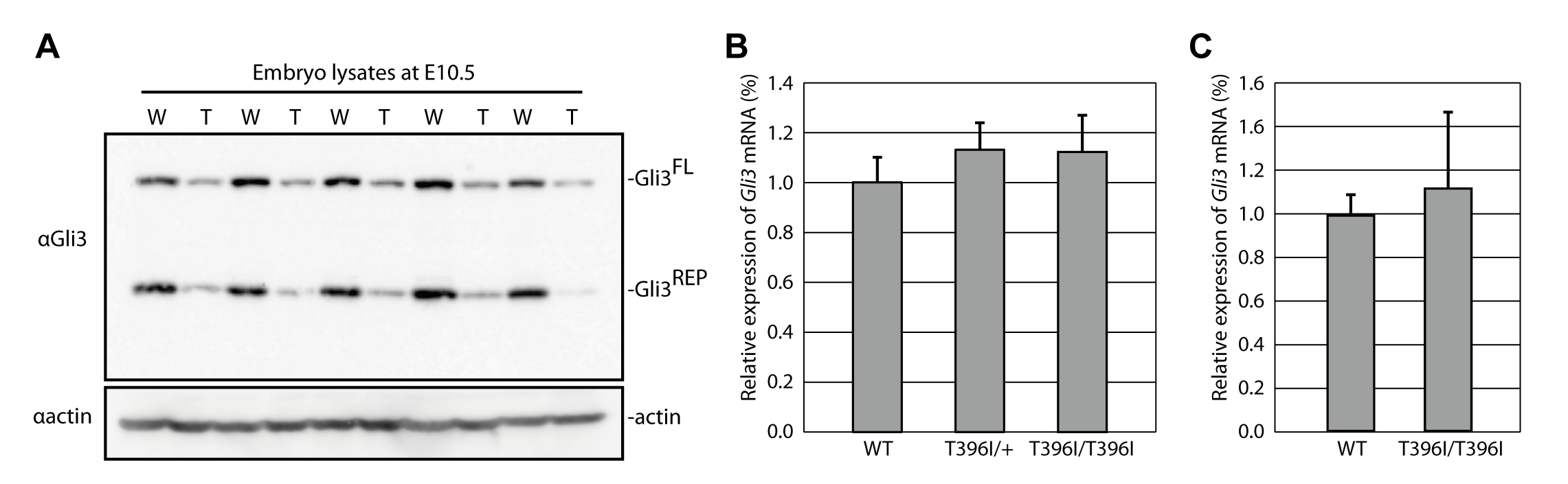

Supplement: S2 Fig — (TIF) [file pone.0119455.s002.tif]

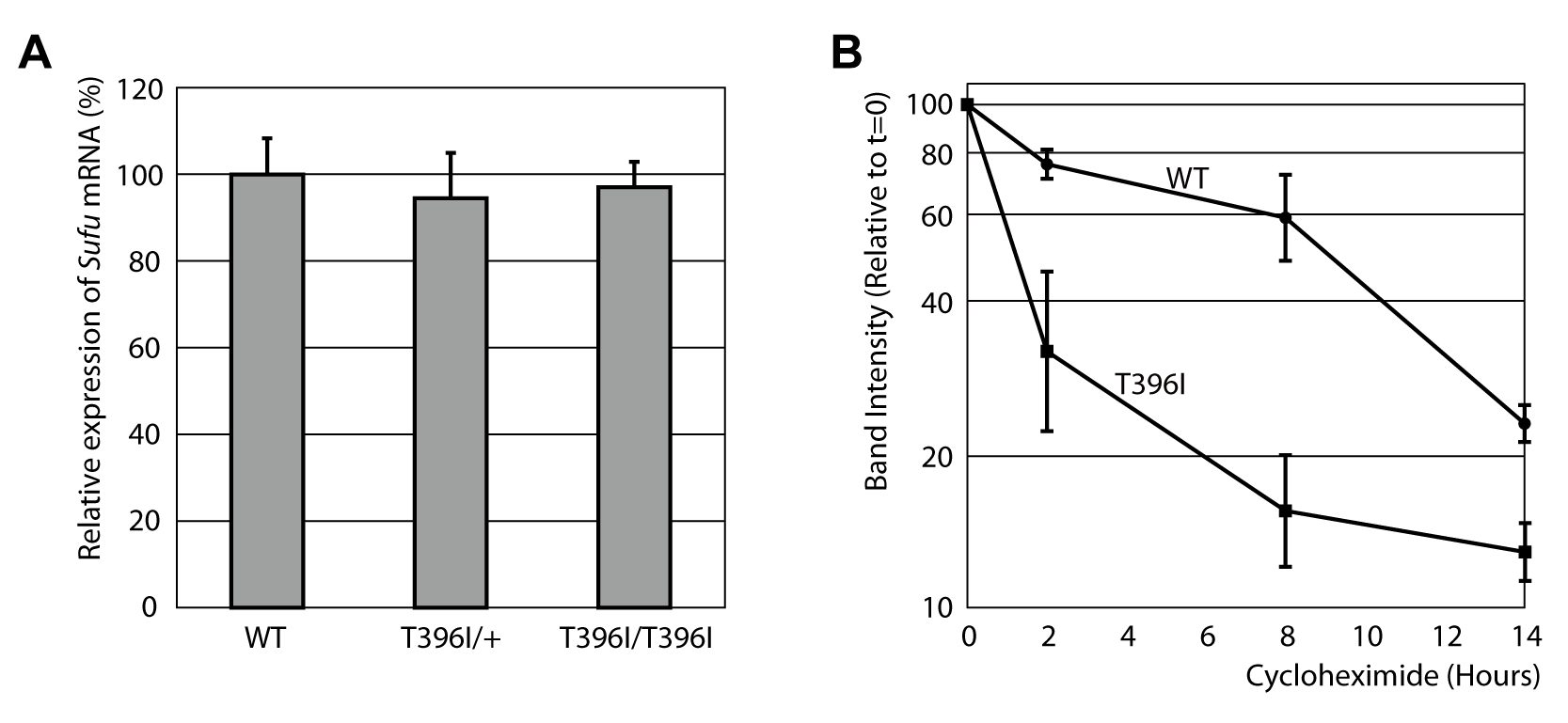

Supplement: S3 Fig — (TIF) [file pone.0119455.s003.tif]

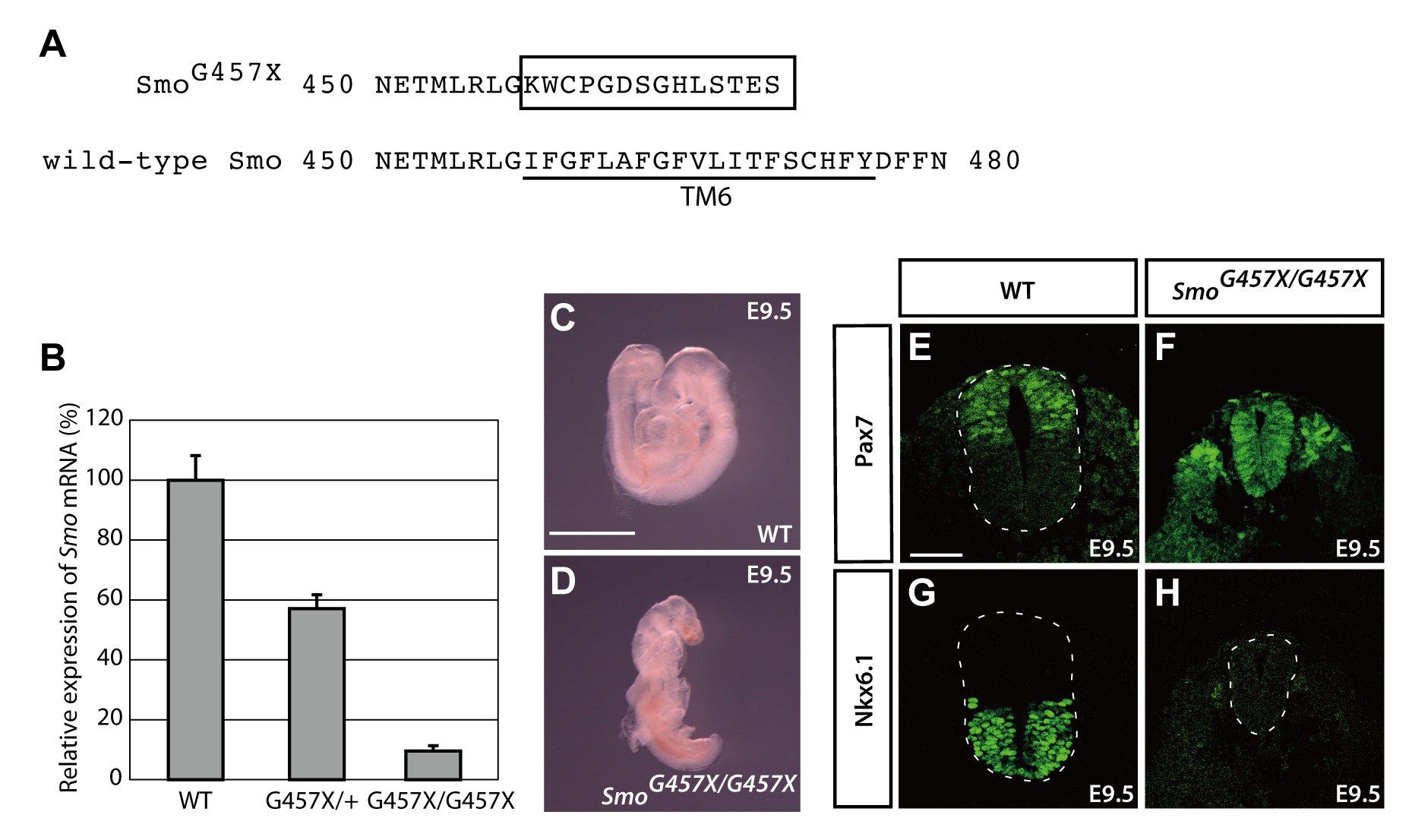

Supplement: S4 Fig — (TIF) [file pone.0119455.s004.tif]

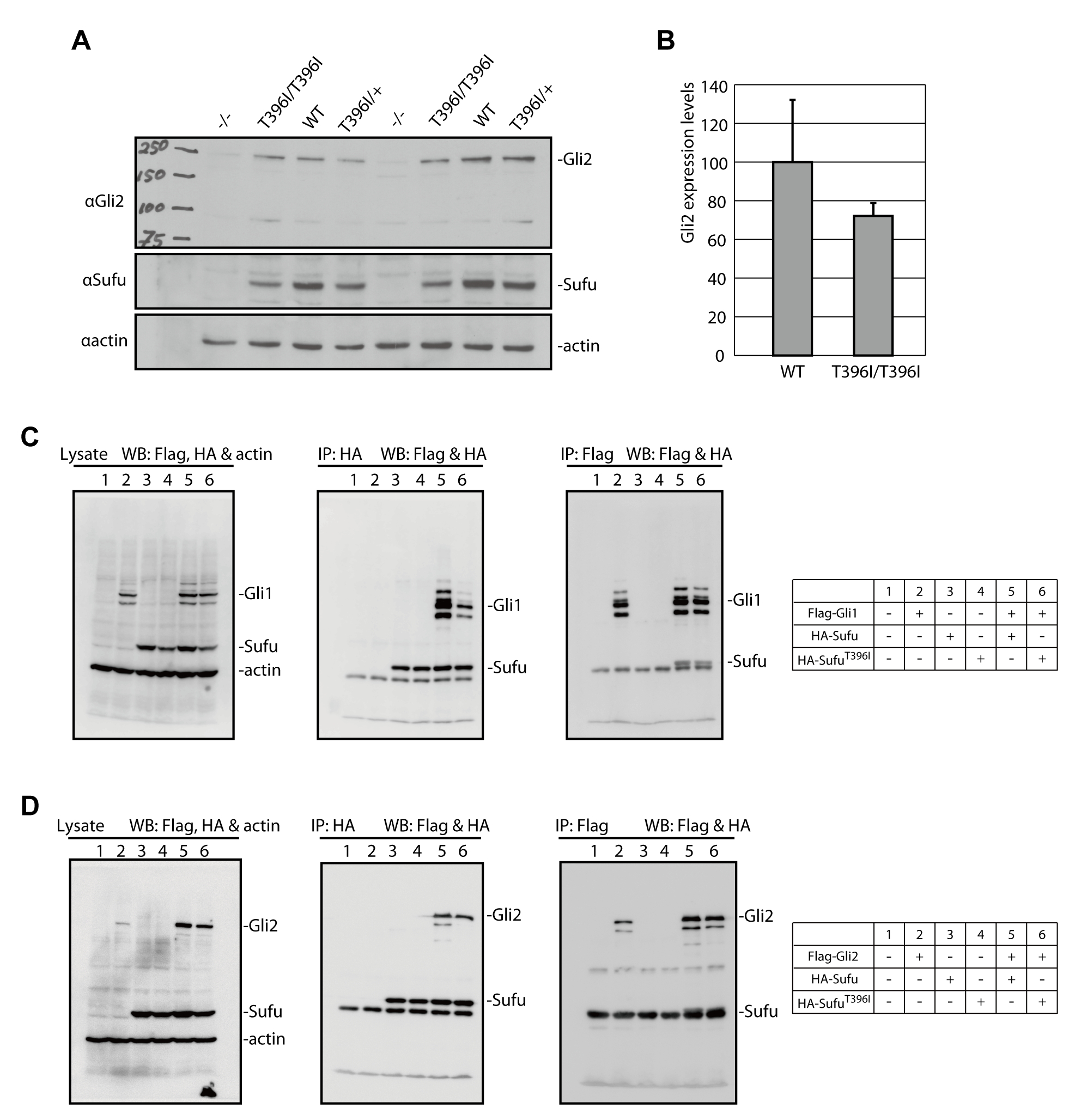

Supplement: S5 Fig — (TIF) [file pone.0119455.s005.tif]

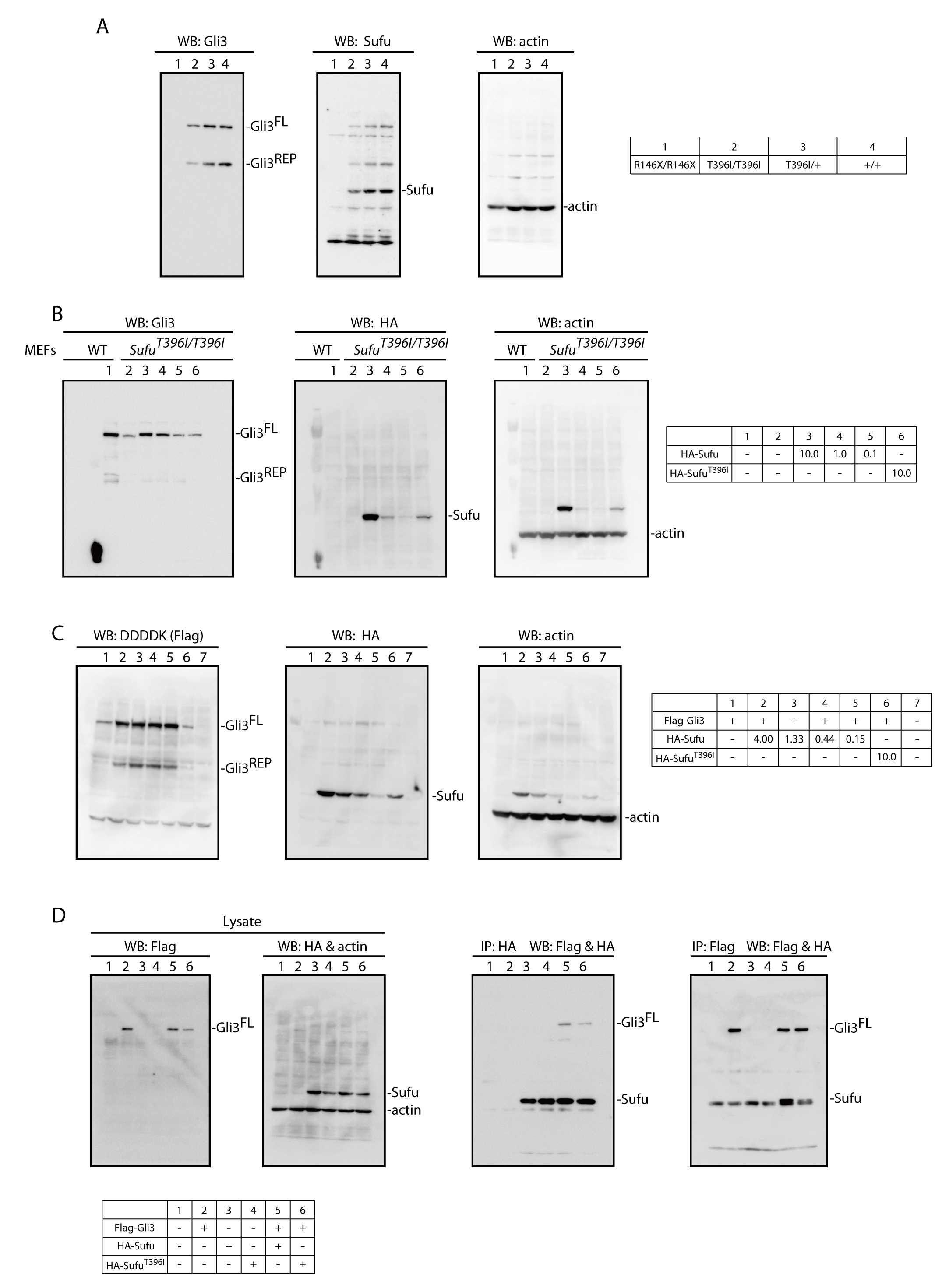

Supplement: S6 Fig — (TIF) [file pone.0119455.s006.tif]

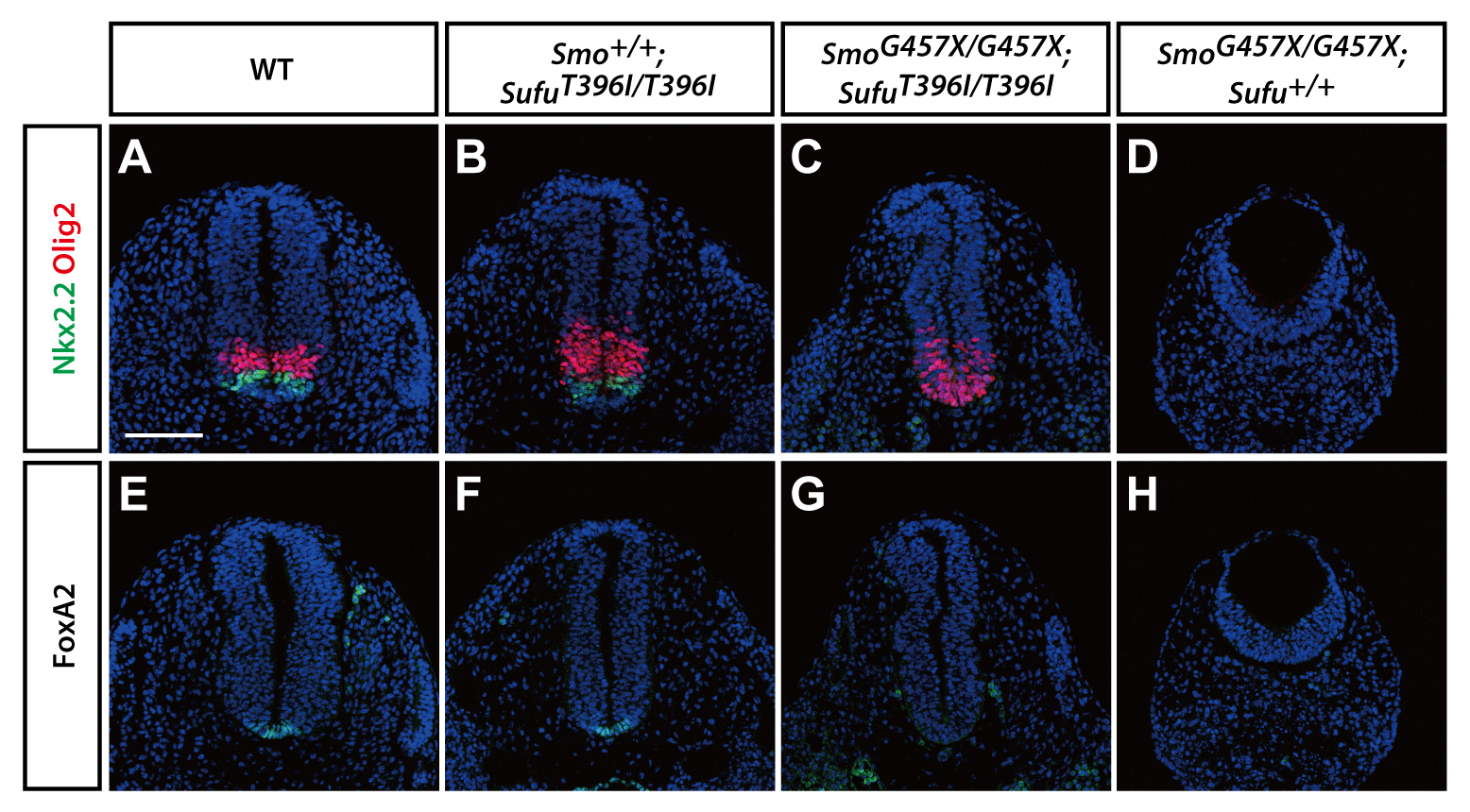

Supplement: S7 Fig — (TIF) [file pone.0119455.s007.tif]

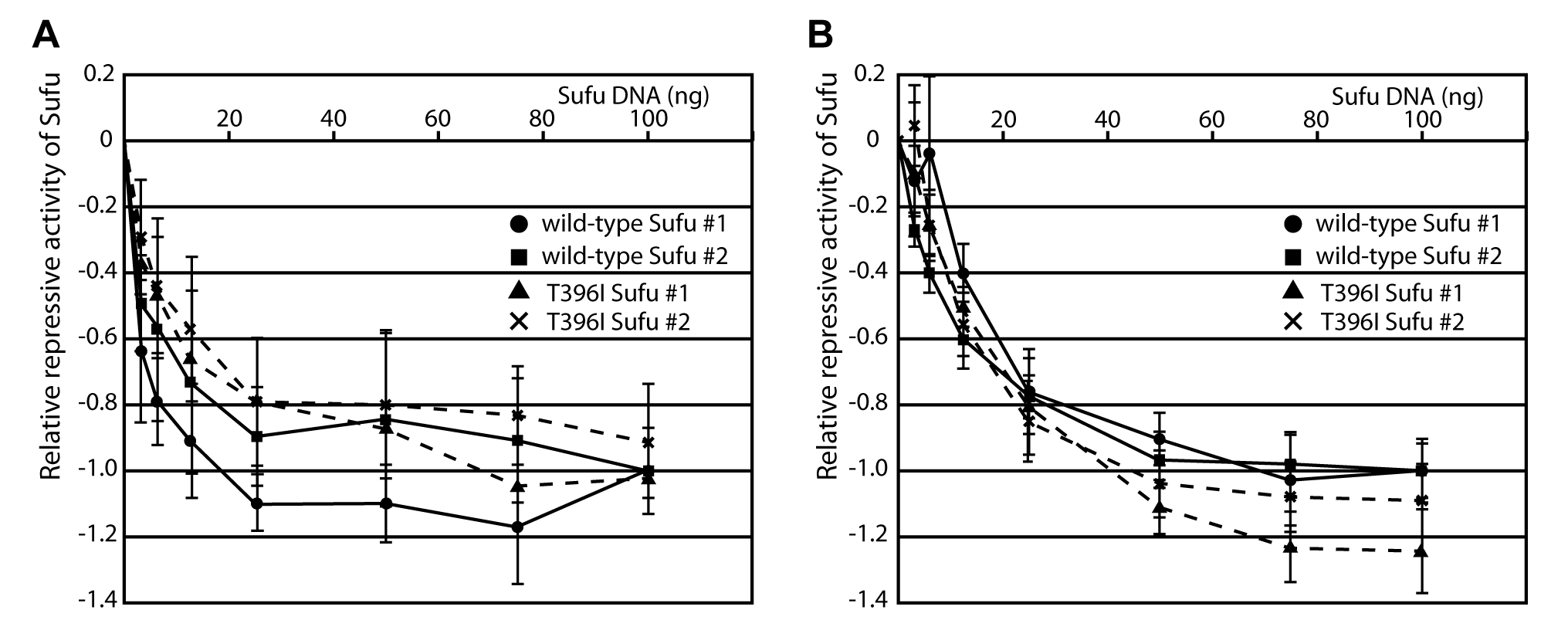

Supplement: S8 Fig — (TIF) [file pone.0119455.s008.tif]
