# Supplementary material for: T396I Mutation of Mouse Sufu Reduces the Stability and Activity of Gli3 Repressor
Source: PLoS One. 2015 Mar 11;10(3):e0119455. doi: 10.1371/journal.pone.0119455 (PMC4356511; doi:10.1371/journal.pone.0119455)
Supplement: S1 File — Table A: Summary of identified mutations for the Sufu and Smo genes. Table B: Quantification of band intensity from Western blotting. Table C: Primer sequences for mutation screening. Table D: Summary of TGCE screening in the Sufu and Smo genes. Table E: Primers used for genotyping by pyrosequencing. Table F: Taqman probes and primer sequences for genotyping. (DOCX) [file pone.0119455.s009.docx]

Supporting Information

**T396I Mutation of Mouse *Sufu* Reduces the Stability and Activity of Gli3 Repressor**

Shigeru Makino^1^*, Olena Zhulyn^2¤^, Rong Mo^2^, Vijitha Puviindran^2^, Xiaoyun Zhang^2^, Takuya Murata^1^, Ryutaro Fukumura^1^, Yuichi Ishitsuka^1^, Hayato Kotaki^1^, Daisuke Matsumaru^3^, Shunsuke Ishii^4^, Chi-Chung Hui^2^, Yoichi Gondo^1^

^1^Mutagenesis and Genomics Team, RIKEN BioResource Center, Ibaraki, Japan

^2^Department of Molecular Genetics, University of Toronto and Program in Developmental and Stem Cell Biology, The Hospital for Sick Children, Toronto, Ontario, Canada

^3^Department of Developmental Genetics, Institute of Advanced Medicine, Wakayama Medical University, Wakayama, Japan

^4^Laboratory of Molecular Genetics, RIKEN Tsukuba Campus, Tsukuba, Ibaraki, Japan

^¤^Current address: Department of Developmental Biology, Stanford University, California, United States of America

* Corresponding author

E-mail: [shmakino@brc.riken.jp](mailto:shmakino@brc.riken.jp) (SM)

Inventory of Supporting Information

Supporting Materials and Methods

Table A. Summary of identified mutations for the *Sufu* and *Smo* genes

Table B. Quantification of band intensity from Western blotting

Table C. Primer sequences for mutation screening

Table D. Summary of TGCE screening in the *Sufu* and *Smo* genes

Table E. Primers used for genotyping by pyrosequencing

Table F. Taqman probes and primer sequences for genotyping

Supporting References

Supporting Figure Legends

S1 Figure. *Sufu^R146X^* is a null allele of *Sufu*

S2 Figure. Expression levels of both Gli3^FL^ and Gli3^REP^ are reduced in *Sufu^T396I/T396I^* embryos

S3 Figure. Stability of the Sufu^T396I^ protein is reduced

S4 Figure. *Smo^G457X^* is a null allele of *Smo*

S5 Figure. Sufu^T396I^ is able to stabilize Gli2 and interact with Gli1 and Gli2

S6 Figure. Full gel images of Western blotting shown in Figure 2

S7 Figure. Nuclear staining of the neural tubes shown in Figure 4

S8 Figure. Qualitatively equivalent activities of wild-type Sufu and Sufu^T396I^ on Gli1 and Gli2 regulations

**Supporting Materials and Methods**

**ENU mouse mutagenesis**

The detailed protocols of the ENU mutagenesis have been described in Inoue et al. [1] and Sakuraba et al. [2] and reviewed in Gondo [3], and can also be found at the Web site <http://www.brc.riken.jp/lab/gsc/mouse/index.html>. In brief, ENU (Sigma) at 85 or 100 mg/kg body weight was intraperitoneally administrated to C57/BL6J males at 8–10 weeks of age, and injections were performed twice with a 1-week interval. Injected males were crossed with DBA/2J or C3H/HeJ females after recovering from an acute sterile period to generate G1 offspring. Sperm from all the G1 males at 3 months of age was cryopreserved and testes were collected for genomic DNA preparation at the same time. Genomic DNA of testes from each G1 male was extracted for the subsequent screening step. C57BL/6J, DBA/2J and C3H/HeJ mice were purchased from CLEA Japan, Inc. (Tokyo, Japan).

**Gene-driven screening**

An outline of gene-driven mutagenesis has been presented in Gondo et al. [4]. Detailed protocols for PCR, heteroduplex formation, and mutation screening using the TGCE system have been previously described [2]. To screen mutations in *Sufu*, PCR primers were generated for all coding exons except the first exon, which included the translation start codon (Table C in S1 File). In addition, we generated primer pairs for the seventh to twelfth exons of *Smo*, corresponding to the C-terminal half of Smo (Table C in S1 File). Screening with the TGCE system, which detects heteroduplexes of mismatch pairs in PCR products, was performed against 6856 G1 mice of the ENU mutagenized mouse genome archive (Table D in S1 File). The site and type of base substitution of the mutations were then confirmed by direct sequencing by ABI3100 sequencer (Life Technologies). As a result, we identified 16 independent mutations each in *Sufu* and *Smo*, seven and six of which altered the Sufu and Smo peptide sequences, respectively (Table A in S1 File).

**Genotyping of mutant mouse strains**

Mice and embryos of *Sufu^T396I^*, *Sufu^R146X^*, and *Smo^G457X^* were genotyped by either direct sequencing, pyrosequencing, or TaqMan assays. Direct sequencing was performed with the same primers used for the gene-driven screening (Table C in S1 File). Pyrosequencing was performed with a PSQ 96 MA system (Pyrosequencing Inc.) using PyroMark Gold Q96 reagents (Qiagen) according to the manufacturer’s instructions. Primers used for pyrosequencing are listed in Table E in S1 File. TaqMan assays were performed on LightCycler 480 (Roche) with TaqMan Genotyping Master Mix (Life Technologies) according to the manufacturer’s instructions. Primers and TaqMan probes are listed in Table F in S1 File.

**Real-time PCR**

Embryos were dissected and stored in RNA Later solution (Life Technologies) until genotyping was completed. Total RNA was isolated with ISOGEN (Nippon Gene) and reverse-transcribed with SuperScript III reverse transcriptase (Invitrogen) and oligo(dT)_20_ primers according to the manufacturer’s protocol. The following primers were used for real-time PCR: Smo, 5′-GCAAGCTCGTGCTCTGGT-3′ and 5′-TCCACTCGGTCATTCTCACA-3′; Sufu, 5′-TCCAGGTTACCGCTATCGTC-3′ and 5′-ACTCAGGCCAAAGCTGATGT-3′; Gli3, 5′- GATCCATCTCCTATTCCTCCA-3′ and 5′- GATCCTAATGAAGGGCAAGTC-3′; GAPDH, 5′-GAGGCCGGTGCTGAGTATGTCGT-3′ and 5′-GGTGGTGCAGGATGCATTGCT-3′. Real-time PCR was performed for at least two embryos from each genotype in triplicate, using LightCycler 480 SYBR Green I Master on a LightCycler 480 system (Roche). Expression levels were normalized against *GAPDH*, and relative expressions compared with those of wild-type embryos were shown.

**Stability of the Sufu protein**

293T cells were seeded in 100-mm dishes and, on the following day, were transfected with plasmids for either HA–Sufu or HA–Sufu^T396I^ expression. Four hours later, cells were trypsinized, and one-twelfth of the cells were seeded in wells of 12-well plates. Twenty-four hours later, cells were changed to low-serum medium (DMEM containing 0.5% fetal bovine serum) and cultured for an additional 12 h. Cells were then treated with 20 μM cychloheximide for 2, 8, and 14 h. Cells were harvested and lysed in lysis buffer (see Materials and Methods in the main text). The supernatants containing equal amounts of protein were subjected to immunoblotting with rabbit anti-HA (ab9110, Abcam) and mouse IgM anti-actin (Ab-1, Calbiochem) antibodies.

Table A Summary of identified mutations for the *Sufu* and *Smo* genes

| Gene | Target exon(s) | Mutation | Allele name | BRC order# | Amino acid change | Sequence surrounding to the mutation |
| --- | --- | --- | --- | --- | --- | --- |
| *Sufu* | exon2 | T→C | Rgsc02071 | RBRC-GD000163 | L68S | CCGGACCCCTTGGACTATGTT |
| *Sufu* | exon3 | 1 bp deletion | Rgsc02075 | RBRC-GD000164 | R146X | CAGGGCCTAGCCCGATATGTC |
| *Sufu* | exon3 | T→A | Rgsc02068 | RBRC-GD000165 | intron | GCGTTTGGTTTGCATTTGCAT |
| *Sufu* | exon3 | T→C | Rgsc02076 | RBRC-GD000166 | intron | TTTTGATGTCTACAGAGCCCT |
| *Sufu* | exon4 | T→A | Rgsc01969 | RBRC-GD000167 | intron | CTCCTGTCTGTCTCTCCACAG |
| *Sufu* | exon4 | C→A | Rgsc02070 | RBRC-GD000168 | S157R | CCTTCTGTAGCGGGGACCATG |
| *Sufu* | exon5&6 | G→T | Rgsc02030 | RBRC-GD000169 | intron | TATGTCTGCCGATCAATGCAG |
| *Sufu* | exon5&6 | T→C | Rgsc02026 | RBRC-GD000170 | intron | AAAACCTTGCTAGCACACTCT |
| *Sufu* | exon7 | G→A | Rgsc02027 | RBRC-GD000171 | E260K | CAAAGGCATTGAGACAGACGG |
| *Sufu* | exon7 | T→A | Rgsc02029 | RBRC-GD000172 | I291N | AGCCGGAGCATCTGCCTCGGC |
| *Sufu* | exon7 | A→G | Rgsc01185 | RBRC-GD000173 | S290G | GGATAGCCGGAGCATCTGCCT |
| *Sufu* | exon8 | T→A | Rgsc02028 | RBRC-GD000174 | intron | GGCAACTTTGTGATTTCATTG |
| *Sufu* | exon10 | C→T | Rgsc02062 | RBRC-GD000175 | T396I | CGGCACTTCACCTACAAGAGT |
| *Sufu* | exon12 | C→T | Rgsc02061 | RBRC-GD000176 | D476D | TTCTCCCCGACGTGGTGTTCG |
| *Sufu* | exon12 | C→T | Rgsc02077 | RBRC-GD000177 | D480D | TGGTGTTCGACAGTCCACTGC |
| *Sufu* | exon12 | A→G | Rgsc02060 | RBRC-GD000178 | 3'UTR | CACCCATCCCAGGCCCCACCC |
| *Smo* | exon7 | T→C | Rgsc02058 | RBRC-GD000179 | intron | CCAAGCTTTATGTAATTAGTC |
| *Smo* | exon7 | G→T | Rgsc02067 | RBRC-GD000180 | intron | CAAGCTTTATGTAATTAGTCA |
| *Smo* | exon7 | A→C | Rgsc00781 | RBRC-GD000181 | intron | ATAGGAATCCAGCGCCATGGG |
| *Smo* | exon7 | T→A | Rgsc02066 | RBRC-GD000182 | intron | GTCCTGCTAATGTCTCAATTC |
| *Smo* | exon7 | G→A | Rgsc02073 | RBRC-GD000183 | G457X | CTGCGCCTGGGTAAGTGGTGC |
| *Smo* | exon8 | T→G | Rgsc01155 | RBRC-GD000184 | intron | TGAGCCCCGATGTAGCCACCC |
| *Smo* | exon8 | A→G | Rgsc02064 | RBRC-GD000185 | D490G | AGCTTCCGGGACTATGTGCTG |
| *Smo* | exon9 | A→T | Rgsc02059 | RBRC-GD000186 | N497Y | ATGCCAAGCCAACGTGACCAT |
| *Smo* | exon9 | A→G | Rgsc02069 | RBRC-GD000187 | N525D | GGAGAAGATCAATCTATTTGC |
| *Smo* | exon10 | T→C | Rgsc02065 | RBRC-GD000188 | intron | AGGCAGCGGCTGCTCTTGCAC |
| *Smo* | exon10 | T→C | Rgsc02063 | RBRC-GD000189 | D561D | GGCACAGTGATGATGAGCCCA |
| *Smo* | exon10 | G→T | Rgsc00898 | RBRC-GD000190 | L583L | GGCGTGAGCTGCTGCAGAACC |
| *Smo* | exon10 | A→T | Rgsc02074 | RBRC-GD000191 | Q589L | AACCCGGGCCAGGAGCTCTCC |
| *Smo* | exon10 | T→C | Rgsc02072 | RBRC-GD000192 | intron | ACACCTGACCTCCACAGTACC |
| *Smo* | exon11 | G→T | Rgsc00992 | RBRC-GD000193 | intron | TCTTCTGCAGGAAGGAGGGGT |
| *Smo* | exon11 | T→A | Rgsc01494 | RBRC-GD000194 | V630E | ACCAAGATGGTGGCTCGGAGA |

| Table B Quantification of band intensity from Western blotting | | | | | |  | |  | | |  |  |
| --- | --- | --- | --- | --- | --- | --- | --- | --- | --- | --- | --- | --- |
|  | | | | | |  | |  | | |  |  |
| Fig. 2D | Lane | 1 | 2 | 3 | 4 | | 5 | | | 6 | |  |
|  | Gli3^FL^/actin | 1.38 | 0.27 | 1.00 | 0.56 | | 0.22 | | | 0.27 | |  |
|  | Gli3^REP^/actin | 5.15 | 0.58 | 1.00 | 1.16 | | 1.04 | | | 0.40 | |  |
|  | Sufu/actin | 0.00 | 0.02 | 1.00 | 0.05 | | 0.02 | | | 0.17 | |  |
|  |  |  |  |  |  | |  | | |  | |  |
| Fig. 2E | Lane | 1 | 2 | 3 | 4 | | 5 | | | 6 | | 7 |
|  | Gli3^FL^/actin | 0.40 | 1.00 | 1.07 | 1.09 | | 1.21 | | | 0.18 | | 0.03 |
|  | Gli3^REP^/actin | 0.23 | 1.00 | 1.71 | 1.41 | | 1.10 | | | 0.16 | | 0.01 |
|  | Sufu/actin | 0.03 | 1.00 | 0.61 | 0.38 | | 0.09 | | | 0.32 | | 0.02 |
|  |  |  |  |  |  | |  | | |  | |  |
| Fig. 2F | Lane | 1 | 2 | 3 | 4 | | 5 | | | 6 | |  |
| IP: HA | Flag–Gli3^FL^ | 0.04 | 0.03 | 0.04 | 0.02 | | 1.00 | | | 0.44 | |  |
|  | HA–Sufu | 0.00 | 0.00 | 0.49 | 0.85 | | 1.00 | | | 0.77 | |  |
| IP: Flag | Flag–Gli3^FL^ | 0.01 | 1.35 | 0.03 | 0.02 | | 1.00 | | | 1.13 | |  |
|  | HA–Sufu | 0.00 | 0.00 | 0.00 | 0.00 | | 1.00 | | | 0.43 | |  |
| Lysate | Flag–Gli3^FL^/actin | 0.01 | 0.84 | 0.02 | 0.07 | | 1.00 | | | 0.36 | |  |
|  | HA–Sufu/actin | 0.10 | 0.07 | 0.83 | 0.56 | | 1.00 | | | 0.55 | |  |
|  |  |  |  |  |  | |  | | |  | |  |
| Fig. 5B | Lane | 1 | 2 | 3 | 4 | | 5 | | | 6 | |  |
| IP: HA | Flag–Gli1 | 0.00 | 0.00 | 0.00 | 0.00 | | 1.00 | | | 0.34 | |  |
|  | HA–Sufu | 0.00 | 0.01 | 0.76 | 0.81 | | 1.00 | | | 0.88 | |  |
| IP: Flag | Flag–Gli1 | 0.00 | 0.67 | 0.00 | 0.00 | | 1.00 | | | 0.91 | |  |
|  | HA–Sufu | 0.00 | 0.00 | 0.00 | 0.01 | | 1.00 | | | 0.62 | |  |
| Lysate | Flag–Gli1/actin | 0.01 | 0.74 | 0.01 | 0.01 | | 1.00 | | | 0.74 | |  |
|  | HA–Sufu/actin | 0.06 | 0.08 | 1.10 | 0.51 | | 1.00 | | | 0.48 | |  |
|  |  |  |  |  |  | |  | | |  | |  |
| Fig. 5C | Lane | 1 | 2 | 3 | 4 | | 5 | | | 6 | |  |
| IP: HA | Flag–Gli2 | 0.00 | 0.00 | 0.00 | 0.06 | | 1.00 | | | 0.99 | |  |
|  | HA–Sufu | 0.00 | 0.00 | 0.91 | 0.82 | | 1.00 | | | 1.10 | |  |
| IP: Flag | Flag–Gli2 | 0.00 | 0.48 | 0.01 | 0.00 | | 1.00 | | | 0.90 | |  |
|  | HA–Sufu | 0.00 | 0.00 | 0.00 | 0.00 | | 1.00 | | | 0.60 | |  |
| Lysate | Flag–Gli2/actin | 0.01 | 0.08 | 0.01 | 0.02 | | 1.00 | | | 0.59 | |  |
|  | HA–Sufu/actin | 0.02 | 0.03 | 0.79 | 0.63 | | 1.00 | | 0.60 | | |  |

Table C Primer sequences for mutation screening.

| PCR amplicon | | | Primer sequences 5’-3’ | |
| --- | --- | --- | --- | --- |
| Gene | # of exon | Length (bp) | F | R |
| *Sufu* | 2 | 400 | ctcactctagcttcccctagc | cgagttccaggctgtcca |
| *Sufu* | 3 | 450 | agggtaaagagggctttgga | agcaacaaagcctcagggta |
| *Sufu* | 4 | 552 | gtatgcacaccagcagccta | tcagctgggaagtctgtcct |
| *Sufu* | 5 and 6 | 600 | tgtgtcccctacttaagacacc | gaattcaggcgacagaccat |
| *Sufu* | 7 | 495 | aaccctgttcccagaggagt | tgctcatatatagttcacccaca |
| *Sufu* | 8 | 400 | tctcaagcttgttgaactcgt | ctacagactcggggtccatt |
| *Sufu* | 9 | 461 | ggaggagacagccattgaga | tctccagtttcccactcagg |
| *Sufu* | 10 | 494 | caggtgggccatcaggag | acctgggttcccactgtcac |
| *Sufu* | 11 | 387 | ctcgagttcgtccctctgtc | cttctcaggggttcctctcc |
| *Sufu* | 12 | 600 | tccagtcacctgtcttgtcc | AGGGTGGAGCAGCCTGAC |
| *Smo* | 7 | 561 | tctgtttcaagctttcaccatc | ctatgggaggccacaggaag |
| *Smo* | 8 | 600 | agtgccaggaccgctacc | cctgctcctgtgcattgact |
| *Smo* | 9 | 500 | ggtctagtggtacagctcagtgg | cagctaagcaattagccatcc |
| *Smo* | 10 | 400 | acaggagccctgcattctg | gagtgcctcattccctagtttg |
| *Smo* | 11 | 500 | ggattttgaatgggcactgg | CActgtcagggggacaaag |
| *Smo* | 12 | 607 | gtcccagaacccaagaacag | GATGAGACCCAGAGGTGTGC |

Table D Summary of TGCE screening in the *Sufu* and *Smo* genes

| Gene | Exon | Size of target sequence in bp (a) | No. of screened G1 DNA | Total length of screening in bp (b) | Total No. of identified mutations | No. of mutations identified in the exons |
| --- | --- | --- | --- | --- | --- | --- |
| *Sufu* | exon2 | 361 | 6836 | 2467796 | 1 | 1 |
| *Sufu* | exon3 | 410 | 6836 | 2802760 | 5 (3) c | 3 (1) c |
| *Sufu* | exon4 | 512 | 6834 | 3499008 | 2 | 1 |
| *Sufu* | exon5&6 | 558 | 6785 | 3786030 | 2 | 0 |
| *Sufu* | exon7 | 452 | 6785 | 3066820 | 3 | 3 |
| *Sufu* | exon8 | 359 | 6785 | 2435815 | 1 | 0 |
| *Sufu* | exon9 | 421 | 6785 | 2856485 | 0 | 0 |
| *Sufu* | exon10 | 456 | 6842 | 3119952 | 1 | 1 |
| *Sufu* | exon11 | 347 | 6834 | 2371398 | 0 | 0 |
| *Sufu* | exon12 | 562 | 6842 | 3845204 | 3 | 3 d |
| *Smo* | exon7 | 519 | 6842 | 3550998 | 5 e | 0 |
| *Smo* | exon8 | 562 | 6834 | 3840708 | 2 | 1 |
| *Smo* | exon9 | 459 | 6836 | 3137724 | 2 | 2 |
| *Smo* | exon10 | 359 | 6842 | 2456278 | 5 | 3 |
| *Smo* | exon11 | 461 | 6834 | 3150474 | 2 | 1 |
| *Smo* | exon12 | 562 | 6836 | 3841832 | 0 | 0 |

^a^PCR amplicon length minus primer length. ^b^Size of target sequence multiplied by the number of G1 mice. ^c^Number of identified mutations including redundant mutations (number of independent mutations). ^d^Including one mutation in the 3′ UTR. ^e^Including one mutation in the splicing donor site.

Table E Primers used for genotyping by pyrosequencing

| Mutation | Primer | Sequence 5’-3’ |
| --- | --- | --- |
|  | PCR primer F | CCCAGGGGCAGACTCCTACA |
| *Sufu^T396I^* | 5′ biotinylated PCR primer R | GGCCATGTCGCCTGTGATACT |
|  | pyrosequencing primer | TACATGGCCGGCACT |
|  | PCR primer F | CAGAGCTGATGCAGGGCCTAG |
| *Sufu^R146X^* | 5′ biotinylated PCR primer R | CACAAACCCCTGGCCTATTACC |
|  | pyrosequencing primer | ATGCAGGGCCTAGCC |
|  | 5′ biotinylated PCR primer F | GCAGCCAGCAAGATCAACG |
| *Smo^G457X^* | PCR primer R | TTGGCTCTGTCCCTGAATATTT |
|  | pyrosequencing primer | CCTGGGCACCACTTA |

Table F Taqman probes and primer sequences for genotyping

| Mutation | Primer/probe | Sequence 5’-3’ |
| --- | --- | --- |
|  | PCR primer F | AGATCCCCACTGTCTCCATATTCC |
| *Sufu^T396I^* | PCR primer R | GGCCATGTCGCCTGTGATA |
|  | Taqman probe-VIC (WT) | TCTTGTAGGTGAAGTGC |
|  | Taqman probe-FAM (T396I) | CTCTTGTAGATGAAGTGC |
|  | PCR primer F | AGTCTGCCCCACCAACATG |
| *Sufu^R146X^* | PCR primer R | GGCCTATTACCTGACTGGAAGAC |
|  | Taqman probe-VIC (WT) | AGGGCCTAGCCCGATAT |
|  | Taqman probe-FAM (R146X) | AGGGCCTAGCC-GATAT |
|  | PCR primer F | CCAGCAAGATCAACGAGACCAT |
| *Smo^G457X^* | PCR primer R | GGATTCAGTACTGAGATGTCCAGAGT |
|  | Taqman probe-VIC (WT) | CGCCTGGGTAAGTG |
|  | Taqman probe-FAM (G457X) | CGCCTGGATAAGTG |

**Supporting References**

1. Inoue M, Sakuraba Y, Motegi H, Kubota N, Toki H, et al. (2004) A series of maturity onset diabetes of the young, type 2 (MODY2) mouse models generated by a large-scale ENU mutagenesis program. Hum Mol Genet 13: 1147-1157.

2. Sakuraba Y, Sezutsu H, Takahasi KR, Tsuchihashi K, Ichikawa R, et al. (2005) Molecular characterization of ENU mouse mutagenesis and archives. Biochem Biophys Res Commun 336: 609-616.

3. Gondo Y (2008) Trends in large-scale mouse mutagenesis: from genetics to functional genomics. Nat Rev Genet 9: 803-810.

4. Gondo Y, Fukumura R, Murata T, Makino S (2010) ENU-based gene-driven mutagenesis in the mouse: a next-generation gene-targeting system. Exp Anim 59: 537-548.

5. Cooper AF, Yu KP, Brueckner M, Brailey LL, Johnson L, et al. (2005) Cardiac and CNS defects in a mouse with targeted disruption of suppressor of fused. Development 132: 4407-4417.

6. Svard J, Henricson KH, Persson-Lek M, Rozell B, Lauth M, et al. (2006) Genetic elimination of suppressor of fused reveals an essential repressor function in the Mammalian hedgehog signaling pathway. Dev Cell 10: 187-197.

7. Pospisilik JA, Schramek D, Schnidar H, Cronin SJ, Nehme NT, et al. (2010) Drosophila genome-wide obesity screen reveals hedgehog as a determinant of brown versus white adipose cell fate. Cell 140: 148-160.

8. Zhang XM, Ramalho-Santos M, McMahon AP (2001) Smoothened mutants reveal redundant roles for Shh and Ihh signaling including regulation of L/R symmetry by the mouse node. Cell 106: 781-792.

9. Wijgerde M, McMahon JA, Rule M, McMahon AP (2002) A direct requirement for Hedgehog signaling for normal specification of all ventral progenitor domains in the presumptive mammalian spinal cord. Genes Dev 16: 2849-2864.

**Supporting Figure Legends**

**S1 Figure. *Sufu^R146X^* is a null allele of *Sufu***

(**A–D**) Immunofluorescence images of transverse sections at thoracic level. Sections from wild-type embryos at E10.5 (A and C) and *Sufu^R146X/R146X^* embryos at E9.5 (B and D) were immunostained with anti-Olig2, anti-Nkx2.2, and anti-FoxA2 antibodies. Scale bar, 100 μm. Expression of these marker genes was expanded along the entire D-V axis in *Sufu^R146X/R146X^* embryos, indicating that Hh signaling was elevated in the neural tubes. (**E**) Real-time PCR analysis of *Sufu* mRNA in wild-type and *Sufu^R146X/R146X^* embryos at E9.5. Expression of the *Sufu^R146X^* mRNA was drastically reduced, which may possibly be because of nonsense mediated mRNA decay (NMD). Experiments were performed in triplicate using three embryos for each genotype, and the data presented in the figure indicate the relative expression of *Sufu*. Error bars indicate standard deviations. Combined with our observation that the Sufu^R146X^ protein failed to repress transcriptional activation of the reporter gene by Gli1 and Gli2 (Figure 5D and 5E), *Sufu^R146X^* is equivalent to a *Sufu* knockout allele [5-7].

**S2 Figure. Expression levels of both Gli3^FL^ and Gli3^REP^ are reduced in *Sufu^T396I/T396I^* embryos**

(**A**) Western blotting of lysates prepared from *Sufu^T396I/T396I^* and wild-type embryos with anti-Gli3 and anti-actin antibodies. Lysates were prepared from five wild-type and five *Sufu^T396I/T396I^* embryos at E10.5, and the wild-type (W) and *Sufu^T396I/T396I^* (T) lysates were loaded on a gel in a pairwise manner. This gel image is representative of repeated experiments. Genotypes and antibodies are indicated at the top and left, respectively. Relative expression levels of Gli3^FL^ (Figure 2B) and Gli3^REP^ (Figure 2B and 2C) were quantified from the band intensity of this image. (**B**) Real-time PCR analysis of *Gli3* mRNA from wild-type, *Sufu^T396I/+^* and *Sufu^T396I/T396I^* embryos at E9.5. Experiment was performed in triplicate using three embryos for each genotype, and the data presented in this figure indicate the relative expression of *Gli3*. Error bars indicate standard deviations. (**C**) Real-time PCR analysis of *Gli3* mRNA from wild-type and *Sufu^T396I/T396I^* MEFs prepared from embryos at E13.5. Experiment was performed in triplicate with the same biological samples, and the data presented in this figure indicate the relative expression of *Gli3*. p = 0.69, two-tailed Student’s t-test. Error bars indicate standard deviations.

**S3 Figure. Stability of the Sufu^T396I^ protein is reduced**

(**A**) Real-time PCR analysis of *Sufu* mRNA from wild-type, *Sufu^T396I/+^*, and *Sufu^T396I/T396I^* embryos at E9.5. Experiment was performed in triplicate using three embryos for each genotype, and the data presented in this figure indicate the relative expression of *Sufu*. Error bars indicate standard deviations. (**B**) Time course of degradation of the Sufu protein. Intensity relative to *t* = 0 is plotted, followed by normalization to actin. Error bars indicate standard deviations.

**S4 Figure. *Smo^G457X^* is a null allele of *Smo***

(**A**) Partial amino acid sequence of Smo. The *Smo^G457X^* mutation is a G-to-A substitution at the splicing donor site of exon 7, resulting in premature termination of the protein product after addition of an aberrant 14 amino acid stretch at the C terminal when translated (boxed sequence). Underline indicates the predicted transmembrane domain 6. (**B**) Real-time PCR analysis of *Smo* mRNA in wild-type, *Smo^G457X/+^*, and *Smo^G457X/G457X^* embryos at E9.5. Expression of the *Smo* mRNA was drastically reduced in *Smo^G457X/G457X^*. Experiments were performed in triplicate using three embryos for each genotype. The data presented in this figure indicate the relative expression of *Smo*. Error bars indicate standard deviations. (**C, D**) *Smo^G457X/G457X^* showed embryonic lethality at around E9 with holoprosencephaly, abnormal left–right formation, and growth retardation. Scale bars, 1 mm. (**E–H**) Neural tube sections from E9.5 embryos of wild-type (E, G) and *Smo^G457X/G457X^* (F, H) were immunostained with antibodies against Pax7 (E, F) and Nkx6.1 (G, H). In the homozygous embryos, Pax7 expression domain was expanded to the ventral midline of the neural tube, whereas Nkx6.1 was not observed. Thus, Hh signaling is defective in *Smo^G457X/G457X^*, and its phenotype is identical to those reported in *Smo* knockout embryos [8,9]. Scale bar, 100 μm.

**S5 Figure. Sufu^T396I^ is able to stabilize Gli2 and interact with Gli1 and Gli2**

**(A)** Left half of this image corresponds to the images shown in Figure 5A. Chemiluminescence images were sequentially stained with indicated antibodies, and the membrane was exposed to an X-ray film. Western blotting was performed with two embryos of *Sufu^-/-^*, *Sufu^T396I/T396I^*, wild-type and *Sufu^T396I/+^* at E9.5. **(B)** Relative expression of Gli2 was quantified from the band intensity shown in S4A Figure as relative ratios of Gli2/actin. *p* = 0.49, two-tailed Student’s t-test. Error bars indicate the standard deviations. **(C, D)** Full gel images shown in Figure 5B and 5C. Chemiluminescence images stained with indicated antibodies were sequentially captured with a LAS 3000 imaging system.

**S6 Figure. Full gel images of western blotting shown in Figure 2**

Chemiluminescence images stained with indicated antibodies were sequentially captured with a LAS 3000 imaging system. A, B, C, and D correspond to the images shown in Figure 2A, 2D, 2E, and 2F, respectively.

**S7 Figure. Nuclear staining of the neural tubes shown in Figure 4**

(**A–H**) Immunofluorescence images of transverse sections with TO-PRO3 staining. Images are identical to those in Figure 4. Scale bar, 100 μm.

**S8 Figure. Qualitatively equivalent activities of wild-type Sufu and Sufu^T396I^ on Gli1 and Gli2 regulations**

(A, B) 3T3 cells were transfected with a mixture of each titrated amount of the Sufu^T396I^ construct (100, 75, 50, 25, 12.5, 6.25, 3.125, or 0 ng) with the Flag–Gli1 (A) or the Flag–Gli2 construct (B) (100 ng). Exactly the same titration assay was conducted with the wild-type Sufu construct with the Flag–Gli1 (A) or the Flag–Gli2 construct (B) (100 ng). Two independent experiments of the triplicate-well luciferase assay (see Methods and Materials) were performed (#1 and #2). The effects of Sufu repressor activity on Gli1 and Gli2 were shown as relative repressive activity of Sufu. Relative luciferase activities with each amount of T396I or wild-type Sufu transfection were subtracted by the luciferase activity of the negative control (without Sufu transfection). All the differences were normalized by the luciferase activity with 100 ng wild-type Sufu, and assigned to -1.0. Error bars indicate standard deviations.
